# A member of the CONSTANS-Like protein family is a putative regulator of reactive oxygen species homeostasis and spaceflight physiological adaptation

**DOI:** 10.1093/aobpla/ply075

**Published:** 2018-12-15

**Authors:** Natasha J Sng, Bryan Kolaczkowski, Robert J Ferl, Anna-Lisa Paul

**Affiliations:** 1 Plant Molecular and Cellular Biology, University of Florida, Gainesville, FL, USA; 2 Microbiology and Cell Science, University of Florida, Gainesville, FL, USA; 3 Horticultural Science Department, University of Florida, Gainesville, FL, USA; 4 Interdisciplinary Center for Biotechnology Research (ICBR), University of Florida, Gainesville, FL, USA

**Keywords:** *Arabidopsis thaliana*, B-box domain, CONSTANS-Like, ecotype-specific, microgravity, reactive oxygen species, spaceflight, transcription factor, uncharacterized genes

## Abstract

A feature of the physiological adaptation to spaceflight in *Arabidopsis thaliana* (*Arabidopsis*) is the induction of reactive oxygen species (ROS)-associated gene expression. The patterns of ROS-associated gene expression vary among *Arabidopsis* ecotypes, and the role of ROS signalling in spaceflight acclimation is unknown. What could differences in ROS gene regulation between ecotypes on orbit reveal about physiological adaptation to novel environments? Analyses of ecotype-dependent responses to spaceflight resulted in the elucidation of a previously uncharacterized gene (*OMG1*) as being ROS-associated. The *OMG1* 5′ flanking region is an active promoter in cells where ROS activity is commonly observed, such as in pollen tubes, root hairs, and in other tissues upon wounding. qRT-PCR analyses revealed that upon wounding on Earth, OMG1 is an apparent transcriptional regulator of *MYB77* and *GRX480*, which are associated with the ROS pathway. Fluorescence-based ROS assays show that OMG1 affects ROS production. Phylogenetic analysis of OMG1 and closely related homologs suggests that OMG1 is a distant, unrecognized member of the CONSTANS-Like protein family, a member that arose via gene duplication early in the angiosperm lineage and subsequently lost its first DNA-binding B-box1 domain. These data illustrate that members of the rapidly evolving COL protein family play a role in regulating ROS pathway functions, and their differential regulation on orbit suggests a role for ROS signalling in spaceflight physiological adaptation.

## Teaser

Plants adjust to spaceflight by changing patterns of gene expression. The analysis of how different *Arabidopsis* plants respond to space resulted in the discovery of a previously uncharacterized gene (*OMG1*), which was consistently induced in space. This research revealed that OMG1 belongs to the CONSTANS-Like protein family and is involved in maintaining the reactive oxygen species pathway, which appears to be an important feature of the physiological adaptation of plants to the spaceflight environment. It also shows that spaceflight experiments can be used as a platform to discover novel gene function and provide further insights into terrestrial plant biology.

## Introduction

The complex networks and signalling pathways that equip plants to cope with environmental challenge developed over evolutionary time in terrestrial environments. Exposing plants to novel environments presents opportunities to understand how plants adjust to conditions outside their evolutionary experience. Spaceflight is one such novel environment, and understanding how plants function outside of Earth’s boundaries is also integral to space exploration. Plants respond to spaceflight in ways that are dependent on species, ecotype, genetics and even organs (Cowles *et al.* 1984; Hoson *et al.* 2002; Soga *et al.* 2002; Paul *et al.* 2013a; Ferl *et al.* 2014). Indeed, even a single gene difference can influence the spaceflight response (Paul *et al.* 2017), thus investigating ecotype-dependent spaceflight transcriptomes can reveal strategies for physiological adaptation to spaceflight while expanding understanding of gene function in terrestrial environments.

The physiological impact of spaceflight is reflected in the patterns of gene expression. Irrespective of plant growth hardware, duration in space or age of plants, one consensus is that spaceflight elicits responses from pathways associated with cell wall remodelling, cell polarity, phytohormone-mediated processes, as well as general stress responses, such as wounding, pathogen defence, heat shock and reactive oxygen species (ROS) (Joo *et al.* 2001; Paul *et al.* 2005, 2012b, 2013a; Correll *et al.* 2013; Zupanska *et al.* 2013; Ferl *et al.* 2014; Mazars *et al.* 2014; Sugimoto *et al.* 2014; Zhang *et al.* 2014; Kwon *et al.* 2015; Schüler *et al.* 2015). In addition, a large proportion of genes differentially expressed in spaceflight encode unknown proteins.

Spaceflight-grown *Arabidopsis* roots tend to be smaller, have fewer lateral roots and shorter root hair development than their terrestrial controls (Paul *et al.* 2012a; Kwon *et al.* 2015). Aspects of these morphologies in ecotype Columbia (Col-0) suggest a relationship between the down-regulation of several peroxidase genes in spaceflight (Kwon *et al.* 2015).

In this study, a spaceflight-induced gene of unknown function identified from roots was investigated for its potential involvement in ROS-related functions. AT1G05290, which we have dubbed *Orbitally Manifested Gene 1* (*OMG1*), was one of the highest significantly (*P* = 0.0005) induced genes in the Advanced Plant Experiment 01 (APEX01) spaceflight data set (fold change = 12) (Paul *et al.* 2013a). Two distinct biochemical assays typically used to measure ROS activity indicated that knocking out OMG1 alters the production of ROS. Co-expression networks around *OMG1* show correlations to genes involved in ROS-associated oxidative phosphorylation pathways Obayashi *et al*. 2014. *OMG1* was also expressed in organs known to highly utilize ROS during development, such as root hairs and pollen tubes (Shin and Schachtman 2004; Potocký *et al.* 2007; Winter *et al.* 2007; Lassig *et al.* 2014). In addition, *OMG1* is an unrealized member of the CONSTANS-Like family of transcription factors. These findings, originating with the novel environment of spaceflight, further the understanding of plant growth and development and provide insight into the role of a heretofore unknown gene.

## Materials and Methods

### ROSMETER analysis

The ROSMETER tool assesses the ROS-related transcriptomic signature in a large-scale *Arabidopsis* gene expression data set (Rosenwasser *et al.* 2013). *Arabidopsis* gene expression profiles from two independent spaceflight experiments, APEX01 and Advanced Plant Experiment 03-2 (APEX03-2), along with their corresponding ground controls, were used to correlate differential gene expression in spaceflight with specific ROS species and origins. The APEX01 experiment utilized two ecotypes and both were planted on each plate of the experiment. Since the entire plate was harvested on orbit to a single tube, the subsequent transcriptomic analyses comprised 60 % Wassilewskija (WS) and 40 % Col-0 (Paul *et al.* 2013b). In the APEX03-2 data set, WS and Col-0 were kept on separate plates, harvested into separate tubes and analysed individually. A data set from Col-0 compared to WS vertically grown in standard laboratory conditions was included as an additional control (Schultz *et al.* 2017). From each data set, Affymetrix Probeset-ID, Gene Locus ID, *P*-value and fold change were listed and submitted to ROSMETER. The heatmap was constructed using the GENE-E software with hierarchical clustering using the Matrix value offered in the software.

### Plant materials and growth conditions

Seeds from both Col-0 and WS were prepared for APEX01 and APEX03-2 as previously described (Paul *et al.* 2012b, 2013b; Sng *et al.* 2014; Ferl and Paul 2016). Briefly, plants were cultured on Phytagel™ 10 cm Petri plates and grown either in the Advanced Biological Research System (ABRS) hardware (APEX01), or in the Veggie hardware (APEX03-2) under continuous lighting of ~80–135 μmol m^−2^ s^−1^ PAR. In this study, the effects of the spaceflight environment were evaluated, which includes ambient radiation, microgravity and attending effects on convection. In both experiments, the ground control plants were grown with a 24- to 48-h schedule delay that allowed the comparable ground growth chambers to be programmed with the precise environmental conditions that the International Space Station (ISS)-grown plants received on orbit.

Plants grown for additional laboratory analyses were grown on Phytagel™ 10 cm plates or in soil, and placed in a greenhouse growth chamber on a 24-h light cycle (~80 μmol m^−2^ s^−1^ PAR) at 22 °C.

### OMG1 promoter::GUSmgfp5 transgenic plants and analyses

A 0.8-kb 5′ flanking region upstream of the OMG1 (AT1G05290) initial Met was amplified from *Arabidopsis* gDNA using primer pair A **[see****Supporting Information—Table S1****]**, which contains restriction enzyme sites BamHI in the forward primer and NcoI in the reverse. The sequenced and confirmed BamHI/NcoI fragment containing the OMG1 5′ flanking region was cloned into pCAMBIA1303 to create the OMG1p::GUSmgfp5 fusion construct. Although this region may not represent the full complement of motifs that regulate OMG1, the 0.8-kb 5′ flanking region does serve to promote OMG1 transcription and will be referred to as the basal promoter. The construct was then transformed into *Agrobacterium* (*Agrobacterium tumefaciens*) strain GV3101 for floral dip transformation into WS plants (Clough and Bent 1998). Ten independent OMG1p::GUSmgfp5 transgenic lines grown on selection media (250 mg L^−1^ Carbenecillin and 50 mg L^−1^ Hygromycin) were screened for T3 homozygosity. Plants grown on Phytagel plates were used for histochemical localization of β-glucuronidase (GUS) (Jefferson *et al.* 1987).

Pollen and pollen tube analyses were performed as described (Li *et al.* 1999). T1 heterozygous populations of pollen grains and tubes were incubated with X-Gluc to reveal the specificity of the histochemical localization in transformed pollen. Basal OMG1 promoter activity in plants requires ~3 h of staining before detection of GUS in the various locations, whereas GUS activity induced by wounding was observed within 30 min of staining.

In the GUS qRT-PCR wounding experiments with OMG1p::GUSmgfp5, comparable leaves from three individual plants were crimped with forceps and left intact on the plant for the duration of each time point (30 min, 1, 4 and 16 h). Leaves were harvested to liquid nitrogen and stored at −80 °C until RNA extraction. In the mock and immediate time point, leaves were either unwounded or crimped and immediately frozen in liquid nitrogen. Total RNA was extracted using RNAeasy (Qiagen) according to the manufacturer’s instructions. cDNA transcribed from total RNA was used to analyse the transcript abundance of *GUSA*. Primer pairs B and C **[see****Supporting Information—Table S1****]** were used for the analyses. *GUSA* transcript abundance was normalized to UBQ11 (AT4G05050).

### Gene expression analyses of OMG1 and other downstream genes

OMG1 RT-qPCR analyses used TaqMan™ (Applied Biosystems) on spaceflight material from APEX01 and APEX03-2. *OMG1* gene expression was normalized to *UBQ11* primer and probe sequences D and E **[see****Supporting Information—Table S1****]**. The *omg1* knockout (KO) line (SALK_045742C) was validated for a homozygous T-DNA insertion using primers F. Homozygous lines were selfed until all progeny were homozygous and screened for the absence of full-length *OMG1* transcript **[see****Supporting Information—Fig. S1****]**. RT-qPCR analyses of *OMG1* expression induced by a time course wounding assay were performed on wild-type (WT) Col-0, WS and *omg1* lines. Three individual plants with leaves of similar ages and sizes were wounded by crushing between two glass slides. The wounded leaves were left attached to the plant for the allotted times (mock, immediate, 5, 10, 30 min and 1 h) before being harvested into liquid nitrogen and stored at −80 °C. RT-qPCR analysis was conducted using SYBR Green master mix (Applied Biosystems) along with the primer pairs C, D (without probe), G–H and L–R **[see****Supporting Information—Table S1****]**.

### Fluorescent biochemical ROS assays

For the ROS accumulation assay, intact leaves from WT Col-0, *omg1* KO and *rbohd* KO (SALK_070610) were wounded with a 10 µL pipette tip and immediately immersed into the fluorescent probe 5-(and 6)-carboxy-2′,7′-dichloro dihydrofluorescein diacetate (DCF-DA) (Sigma) solution as described in (Beneloujaephajri *et al.* 2013). Briefly, wounded and unwounded leaves were vacuum infiltrated in the dark for 20 min in 60 µM of DCF-DA in standard buffer. Leaves were then rinsed in standard buffer and observed under GFP bandpass filters using the Olympus BX51 compound light microscope at 10× magnification with 55 ms exposure time. Quantification of fluorescence intensity using ImageJ software was previously described (Jakic *et al.* 2017).

A luminol-based ROS assay was done by measuring hydrogen peroxide produced by leaf discs when exposed to flg22. Discs (0.125 cm^2^) of 6-week-old WT Col-0, *omg1* and *rbohd* plants were incubated in 100 µL ddH_2_O overnight in 96-well luminometer plates. Water was replaced with the reaction solution of 40 µM luminol (Sigma-Aldrich) and 10 µg mL^−1^ horseradish peroxidase (Sigma-Aldrich) supplemented with 50 nM of flg22 or water for the mock controls. ROS measurements expressed as means of RLU (relative light units) were taken at 2-min intervals for a period of 38 min.

### Phylogenetic analysis

Protein sequences were identified by rpsblast search of the NR database (Marchler-Bauer and Bryant 2004; Marchler-Bauer *et al*. 2015; NCBI Resource Coordinators 2016); full-length protein sequences containing a single CCT domain (pfam06203) with *e*-value < 0.01 were considered potential OMG1 homologs. Protein sequences were aligned using Clustal Omega v1.2.3 (Sievers *et al*. 2011), PROBALIGN (Roshan and Livesay 2006), MUSCLE v3.8.31 (Edgar 2004), MSAProbs v0.97 (Liu *et al.* 2010), PROBCONS (Do *et al.* 2005) and mafft-einsi v7.215 (Katoh and Standley 2013), with default parameters. Alignments were left unprocessed or processed by Gblocks v0.91 to remove potentially ambiguous regions (Talavera and Castresana 2007). The minimum number of sequences for a flank position (-b2) was 3/5 the total number of sequences. The maximum number of contiguous non-conserved positions (-b3) was 10. The minimum block length (-b4) was 5, and gap positions were allowed (-b5 = a). Other Gblocks parameters were left at default values. Initial maximum likelihood phylogenies were constructed from each alignment using FastTree v2.1.7 with default parameters (Price *et al*. 2010). Initial trees were used as starting trees for full maximum likelihood reconstruction using RAxML v8.2.8 (Stamatakis 2014). Maximum likelihood phylogenies produced from each alignment were converted to a clade presence-absence matrix using the Super Tree Toolkit v0.1.2 (Hill and Davis 2014), and a supertree was inferred from this matrix using the BINCAT model in RAxML (Nguyen *et al*. 2012). Additionally, all individual sequence alignments were concatenated into a single supermatrix, which was used to infer the maximum likelihood protein family phylogeny using RAxML, with the best-fit evolutionary model selected by AIC (Wheeler *et al*. 1995). A consensus of ‘supertree’ and ‘supermatrix’ results was plotted using FigTree v1.4.3.

Protein structural modelling of the B-box2 domain was done using both the Phyre2 and Chimera (Pettersen *et al.* 2004). The closest OMG1 homolog, c2junA of the human Midline-1 (MID1) with a 92 % confidence level was used to predict the overall folding of the B-box domains in OMG1. The predicted structural model of OMG1 was compared to the x-ray crystal structure of c2junA using Chimera and MatchMaker. Side chains positioned near the zinc ions were displayed to predict the potential of OMG1 binding to zinc ions.

### 35S promoter:OMG1cds-sGFPS65T fusion construct for subcellular localization

The coding sequence of OMG1 (RefSeq ID:NP_172021.2) was amplified from cDNA using primer pair I **[see****Supporting Information—Table S1****]**. The PCR fragment was sequenced before being cloned into the pCAMBIA1303 along with sGFP(S65T) (Chiu *et al.* 1996) gene via SpeI and BstEII restriction sites using primer pairs J **[see****Supporting Information—Table S1****]**. Primer pair K was then used to amplify the entire OMG1cds-sGFP(S65T), which was validated by sequencing and cloned into pCAMBIA1302 vector via NcoI and BstEII restriction sites. For subcellular localization, *Agrobacterium*-GV3101 containing 100 µM acetosyringone, 0.05 % Silwet-77 and the pCAMBIA1302 vector or pCAMBIA1302-OMG1cds-sGFPS65T fusion vector were incubated in the dark for 20 min at 28 °C. Leaves of 4-week-old tobacco (*Nicotiana benthamiana*) seedlings were injected and GFP signal was observed after 2 days at 25 °C in the dark. DAPI was used to indicate the nucleus.

## Results

### ROS-producing genes are differentially expressed in response to spaceflight in an ecotype-specific manner

**Figure 1.**
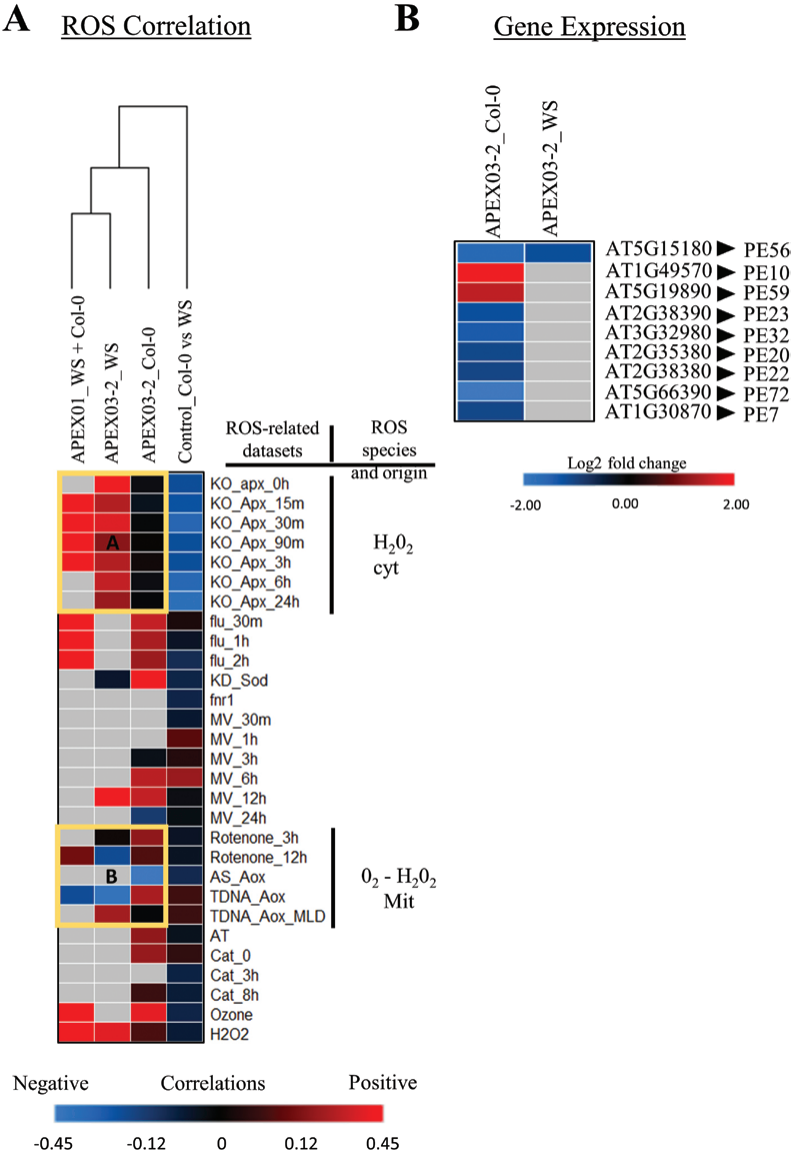
Correlations between spaceflight transcriptome and ROS-related microarrays revealed that ROS is induced in spaceflight in an ecotype-dependent manner. (A) The microarray (APEX01) and the RNAseq (APEX03-2) analyses of spaceflight transcriptional profile relative to the ground control profile were correlated to known ROS-related data sets producing specific ROS species from specific locations within the cell using the bioinformatics tool called ROSMETER (Rosenwasser *et al.* 2013). Positive correlations (red) show ROS production, whereas negative correlations (blue) show ROS scavenging. Black cells suggest no correlations, whereas gray cells indicate that there are insufficient gene numbers for a statistical correlation. Clusters highlighted in yellow boxes denote regions of interest. Group A showed that only the spaceflight transcriptome of WS positively correlated with the cytoplasmic ascorbate peroxidase knockout (KO-*APX1*) experiments, which represent cytoplasmic H_2_O_2_ ROS indices. Group B showed ecotype-related differences in the correlations associated with mitochondrial stress. (B) Peroxidase genes having statically (*P* < 0.01) differential expression (>1 or −1 < log2 fold) between spaceflight and ground control in both WS and Col-0 ecotypes are depicted. Blue is indicative of down-regulation, red is indicative of up-regulation and gray indicates no statistically significant differential expression.

The differential gene expression data sets of the three independent spaceflight experiments and their comparable ground controls were used in the ROSMETER analyses. The heatmap shows the positive-(red), negative-(blue) and no correlation-(black) between the ROS indices and the queried spaceflight transcriptomes (Fig. 1A). A general positive correlation of ROS-producing genes was observed in all the spaceflight transcriptomes and hierarchical clustering displayed a higher similarity with the APEX03-2_WS data set compared to the APEX03-2_Col-0 data set. Two interesting ROS correlation groups were observed in the heatmap. Group A showed that only the spaceflight transcriptome of WS positively correlated with the cytoplasmic ascorbate peroxidase knockout (KO-*APX1*) experiments, which represent cytoplasmic H_2_O_2_ ROS indices, whereas in the Col-0 background cytosolic ROS shows no correlation. Group B showed ecotype-related differences in the correlations associated with mitochondrial stress. Specifically, TDNA-AOX-MLD and Retenone-3h indices showed that if one ecotype had a positive correlation the other had no correlation. In addition, in the Retenone-12h and TDNA-AOX indices, the mixed ecotype transcriptome shows a similar trend to one of the two ecotypes. The control data set, which reflects terrestrial ecotype differences between Col-0 and WS, showed that many of the genes differentially expressed between the two ecotypes are associated with the ROS-scavenging mechanism. A representative transcriptome heatmap from the APEX03-2 spaceflight experiment that compared several significantly (*P* < 0.01) differentially expressed (>1 or −1 < log2 fold change) peroxidase genes between 4-day-old Col-0 and WS ecotypes illustrated that Col-0 alters the expression of many more peroxidase genes in spaceflight than does WS (Fig. 1B). Peroxidases, an integral part of ROS signalling in cell wall remodelling, cell elongation, auxin catabolism, and response to abiotic and biotic stress, (Shigeto and Tsutsumi 2016) were found to be differentially expressed in spaceflight.

### OMG1 transcript is up-regulated in WS ecotype across multiple spaceflight experiments

**Figure 2.**
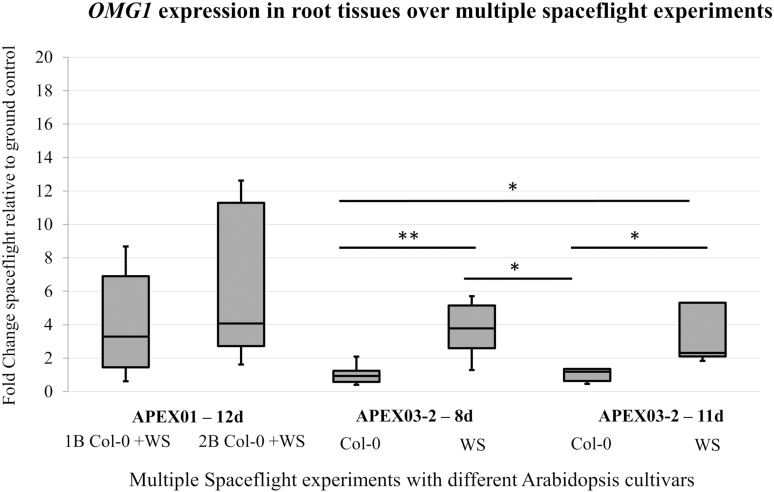
OMG1 transcripts are up-regulated in WS but unchanged in Col-0 consistently over multiple spaceflight experiments. Ecotypes WS or Col-0 were grown onboard the ISS for the number of days (d) indicated, harvested and compared to comparable ground controls. OMG1 transcripts for flight and ground control samples assayed with qRT-PCR. Spaceflight experiment APEX01-2B launched to the ISS via STS131 and analyses published in Paul *et al.* (2013b). The same RNA used in the 12d APEX01 microarrays was used for qRT-PCR shown here. Spaceflight experiment APEX01-1B launched to the ISS via STS129, and spaceflight experiment APEX03-2, 8 day and 11 day was launched to the ISS via SpaceX05. Boxplots were constructed from nine replicates for each sample. Expressions were normalized to UBQ11 and plotted spaceflight relative to ground control using results generated from TaqMan™ qRT-PCR. Spaceflight experiments show that OMG1 transcript is up-regulated in spaceflight relative to ground controls in WS plants, whereas in Col-0 it remains largely unchanged. Significance *(*P* < 0.01), ** (*P* < 0.001) between samples is calculated by doing a single-factor ANOVA followed by *post hoc* Student’s *t*-test with Bonferroni correction.

*OMG1* expression was up-regulated in WS roots across multiple spaceflight experiments (APEX01-1B, APEX01-2B and APEX03-2) but remained unchanged in Col-0 (Fig. 2). The Box-Whisker plot of Fig. 2 illustrates the median fold change of *OMG1* expression between spaceflight and the ground control from a total of nine replicates from each sample. Each spaceflight experiment represents a distinct launch and orbital plant growth event. In both the APEX01 experiments, seeds germinated on orbit grew for 12 days before being harvested. *OMG1* expression shows a 4-fold change in spaceflight compared to ground controls in APEX01-2B and 3.5-fold change in APEX01-1B. In both analyses, the large spread between the maximum and the minimum can be attributed to the mixture of ecotypes. In a subsequent spaceflight experiment, APEX03-2, plants grew for 8 or 11 days on the ISS before being harvested. In the APEX03-2 8-day-old plants, Col-0 showed a median fold change of 1 (no change), whereas WS showed a 3.9-fold increase in spaceflight. In the APEX03-2 11-day-old plants, Col-0 showed a median fold change of 1 and WS showed a 2.1-fold increase in expression.

### The OMG1 basal promoter activity was observed in roots, pollen tubes and upon wounding

**Figure 3.**
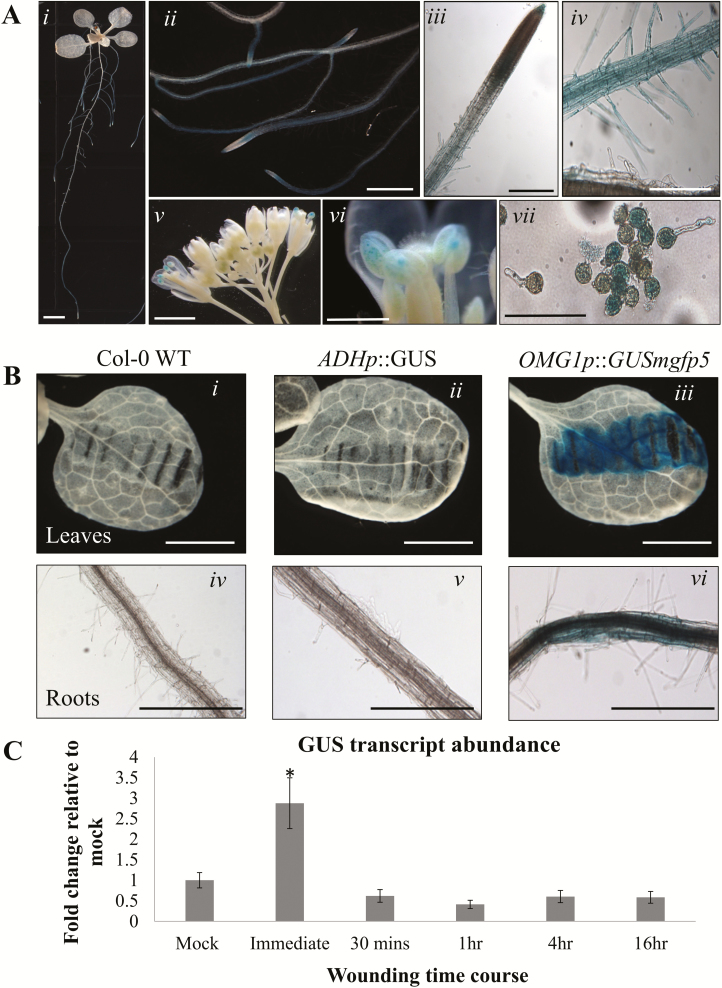
A transgenic reporter line *OMG1* promoter::GusGFP in the WS ecotype background shows OMG1 5′ flanking region activity. (A) Transgenic *Arabidopsis* plants of various ages underwent histochemical GUS staining with X-Gluc. The images show representative distribution patterns of GUS staining in (i) maturation zone of roots, (ii–iii) lateral roots, (iv) root hairs of the maturation zone, (v–vii) pollen and pollen tubes. Scale bars: (i–ii) 1 mm; (iii–vii) 100 µm. (B) OMG1 5′ flanking region activity was also observed upon wounding with a pair of forceps. Wild-type (WT) Col-0, *Adh* promoter::Gus transgenic line were used as a control to ensure GUS enzymatic activity was specific for *OMG1* 5′ flanking region. Both leaf (i–iii) and root tissue (iv–vi) from the various lines were used. Scale bars: 100 µm. (C) Intact leaf tissues were wounded and left for the allotted times before they were harvested for qRT-PCR analysis. The GUS transcript abundance fold change relative to mock was done to access the rapid wounding response of OMG1 5′ flanking region. All error bars represent the standard error of the mean (SEM) of triplicate representative experiments. * indicate significant (*P* < 0.05) difference between immediate and the various wounding time points by doing a single-factor ANOVA followed by *post hoc* Student’s *t*-test with Bonferroni correction.

Histochemical GUS staining revealed some basal *OMG1* promoter activity in roots and pollen but showed heighten activity upon wounding in the roots and leaves. T3 homozygous *OMG1*p::*GUSmgfp5* transgenic plants were used to characterize the activity of the *OMG1* promoter. In roots, basal *OMG1* promoter activity was observed at low levels in the maturation zone of both the primary and lateral roots and root hairs (Fig. 3A, i–iv). In addition, basal promoter activity was also observed in pollen grains and pollen tubes (Fig. 3A, v–vii). A T1 heterozygous population of pollen grains grown in pollen germinating media (Fig. 3A, vii) was used to visualize the specificity of the GUS enzymatic reactions in transformed pollen. Serendipitously, wounded plant tissues were observed to have heightened *OMG1* promoter activity (Fig. 3B).

To ensure that *OMG1* promoter activity was specific to wounding, leaf and root tissues from WT Col-0, *ADHp::GUS* (Chung and Ferl 1999) and *OMG1p*::*GUSmgfp5* lines were wounded with a pair of forceps. In this wounding experiment, GUS staining was observed only in the *OMG1p::GUSmgfp5* line. A qRT-PCR analysis of *GUSA* transcripts from leaves wounded at different time points showed that *OMG1* promoter activity heightens immediately after wounding and dissipates after 30 min (Fig. 3C).

### OMG1 regulates the expression of MYB77 and GRX480 known to be induced in spaceflight and upon wounding

**Figure 4.**
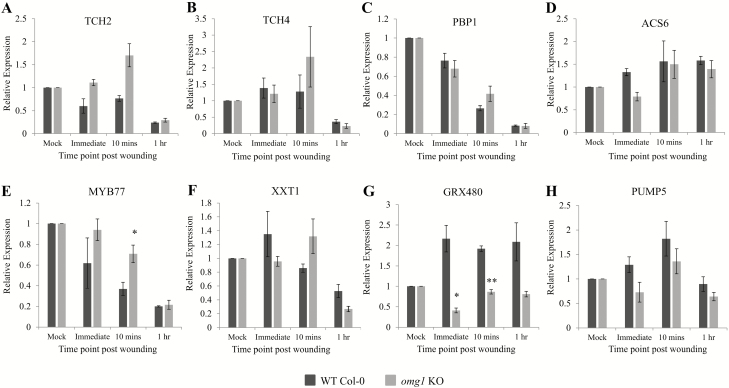
*OMG1* regulates the rapid wounding response of *MYB77* and *GRX480*. WT Col-0 and *omg1* KO intact leaf tissues were untouched (mock) or wounded with a pair of forceps and left for the indicated time points before harvesting for qRT-PCR analyses. Expression of the indicated genes (A–H) shared between spaceflight, rapid wounding and pollen development **[see****Supporting Information—Table S2****]** in the WT (Col-0) and *omg1* KO leaves was analysed. Untouched leaves (mock), immediately after wounding, 10-min post-wounding and 1-h post-wounding were the indicated time points that the transcripts were measured. The average relative expression was normalized to a housekeeping gene (UBQ11) and presented relative to the mock controls. * indicate significance (*P* < 0.05), ** (*P* < 0.001) between WT Col-0 and *omg1* which was calculated by doing a two-factor ANOVA followed by *post hoc* Student’s *t*-test. All error bars represent the standard error of the mean of triplicate representative experiments.

**Figure 5.**
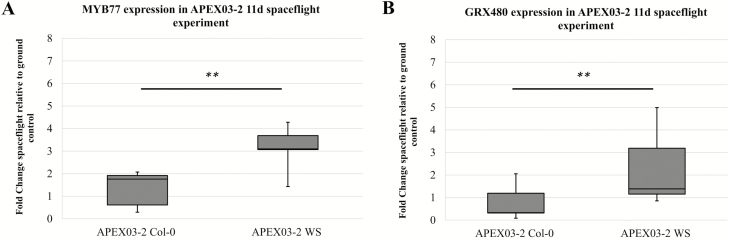
*MYB77* and *GRX480* expression of APEX03-2 spaceflight experiment showed higher fold change in WS compared to the Col-0 ecotype. (A) qRT-PCR done on APEX03-2 11d spaceflight root tissue show that *MYB77* transcript in spaceflight relative to ground controls is significantly (*P* = 0.001) different between WS and Col-0 plants. The fold change of *MYB77* expression is higher in the WS ecotype compared to the Col-0 ecotype. (B) qRT-PCR showed a significant (*P* = 0.007) difference in the expression fold change of *GRX480* between Col-0 and WS plants. Boxplots were derived from nine replicates normalized to UBQ11 and plotted spaceflight relative to ground control. ** indicate (*P* < 0.01) significant difference between Col-0 and WS was calculated by doing a Student’s *t*-test.

Eight genes **[see****Supporting Information—Table S2****]** were used to elucidate potential functional interactions with OMG1. Since *OMG1* promoter activity was shown to rise immediately after wounding (Fig. 3C), a wounding time course of mock, immediate, 10 min and 1 h was used to screen the expression of the genes listed in **Supporting Information—Table S2** that could be affected by knocking out *OMG1*. Two genes, *GRX480* (At1g28480) and *MYB77* (At3g50060), out of eight genes tested showed significant differences (Student’s *t*-test *P* < 0.05) in gene expression between WT and *omg1* lines (Fig. 4D and F). The expression of *GRX480* was repressed after wounding in the *omg1* line and did not increase as did the WT line. On the other hand, *MYB77* expressed in the *omg1* line showed a significant delay in down-regulating gene expression when compared to the WT at the 10-min post-wounding time point. MYB77 and GRX480 were significantly differentially expressed between Col-0 and WS (Fig. 5). Col-0 showed a median fold change of 1.7 and WS showed a 3-fold increase in *MYB77* expression in spaceflight relative to ground controls (Fig. 5A). In the *GRX480* analyses, Col-0 showed a median fold change of 0.3 and WS showed a median fold change of 1.3 in spaceflight relative to ground controls (Fig. 5B).

### ROS accumulation upon wounding and flg22 induction is altered in *omg1* KO leaves compared to Col-0 leaves

**Figure 6.**
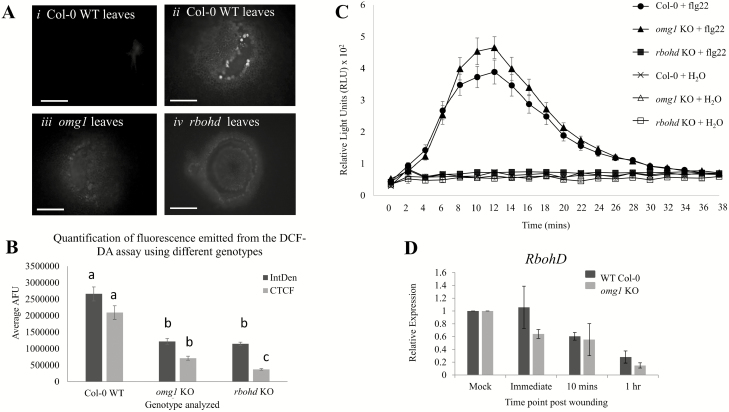
Reactive oxygen species (ROS) accumulation upon wounding and flg22 induction is altered in *omg1* leaves compared to Col-0 leaves. (A and B) DCF-DA ROS assay performed on wounded leaf tissues reveals that OMG1 affects cellular ROS production. (A) DCF-DA is a fluorogenic dye that measures hydroxyl, peroxyl and other ROS activity within the cell. A pipette tip was used to wound the surface of the leaves from the WT line, *omg1* KO line and *rbohd* KO line (a known ROS-deficient mutant). Upon wounding, ROS is usually produced around the site of injury. The DCF-DA reagent is then oxidized by ROS resulting in appearance of green fluorescence. Plants were wounded and immediately placed in the DCF-DA solution for 20 min before visualized on the light microscope. (i) WT Col-0 leaves without wounding, (ii) WT Col-0 leaves, (iii) *omg1* leaves and (iv) *rbohd* leaves were placed in DCF-DA solution immediately after wounding. In the WT plants green fluorescence around the wound site indicates the production of ROS. However, in the *omg1* KO line and the *rbohd* KO line, ROS production was not detected. Images were taken on the Olympus BX51 compound scope at 10× magnification, exposure time 55 ms. Scale bars: 100 µm. (B) Quantification of fluorescence was performed using integrated density (IntDen) and corrected total cell fluorescence (CTCF) [CTCF = Integrated density − (Area of selected cell × Mean fluorescence of background readings)] in ImageJ. Statistical analyses were performed using Student’s *t*-test, bar graphs with different letters show significant difference (*P* < 0.01). (C and D) Higher elicitor-induced oxidative burst in *omg1* KO is not associated with altered expression of *RbohD*. (C) Extracellular ROS production was measured using a luminol-based assay in leaf discs from *omg1* KO, *rbohd* KO and WT Col-0 plants after elicitation with water or flg22. Experiments were repeated at least three times and results are a mean of 24 samples. (D) The relative expression of RbohD in the WT (Col-0) and *omg1* KO leaves was analysed in a wounding assay untouched leaves (mock), immediately after wounding, 10-min post-wounding and 1-h post-wounding. The average relative expression was normalized to a housekeeping gene (UBQ11) and presented relative to the mock controls. No significant difference in RbohD expression between Col-0 and omg1 KO plants was observed when the Student’s *t*-test analysis was performed. All error bars represent the standard error of the mean of triplicate representative experiments.

Wild-type Col-0, *omg1* and respiratory burst oxidase homology D (*rbohd*) KO plants, which are delayed in ROS accumulation (Miller *et al.* 2009), were subjected to mechanical wounding with a pipette tip. The production of hydroxyl, peroxyl and other ROS activity within the cells was measured with DCF-DA assay. Green fluorescence at the site of wounding was captured after 20 min with light microscopy. Wild-type Col-0 leaves produced ROS around the wound site, whereas neither *omg1* nor *rbohd* showed prominent production of ROS (Fig. 6A). Quantification of fluorescence using ImageJ showed significant differences (Student’s *t*-test *P* < 0.01) between Col-0 and the mutant lines (Fig. 6B).

Based on luminol ROS assays OMG1 affects the elicitor-induced generation of extracellular ROS. Flg22-induced hydrogen peroxide production by leaf discs of *omg1* mutants was ~20 % higher compared to Col-0. Water-treated controls along with the *rbohd* leaf discs showed no production of flg22-induced H_2_O_2_ (Fig. 6C). Since elicitor-induced extracellular ROS is governed by *RbohD*, the measurement of *RbohD* expression between Col-0 and *omg1* lines in a wounding time course was analysed (Fig. 6D). The relative expression of *RbohD* showed no significant difference between WT and *omg1* leaves.

### OMG1 is a member of the CONSTANS-Like protein family

The predicted *OMG1* gene encodes a 369 amino acid protein that contains a C-terminal 43 amino acid sequence that is 84 % sequence similarity to the (CONSTANS (CO), CONSTANS-Like (COL) and TIMING OF CAB EXPRESSION1 (TOC1)) ‘CCT’ domain (Berardini *et al.* 2015). OMG1 also contains two divergent B-box domains in the N-terminal region **[see****Supporting Information—Fig. S2****]**. The OMG1 B-box1 domain has 51 % similarity to the B-box1 consensus sequence, and the OMG1 B-box2 domain has a 94 % similarity to the B-box2 consensus sequence (Griffiths *et al.* 2003; Masaki *et al.* 2005; Gangappa and Botto 2014).

A consensus phylogeny suggests that OMG1 belongs to group II of the CO and COL family of transcriptional regulators (Fig. 6). Maximum likelihood phylogenies placed a clade of OMG1-related sequences from Brassicas and Monocots (SH-like aLRT > 0.989) close to COL11-12 sequences from across flowering plants **[see****Supporting Information—Table S3****]**. OMG1 appears closely related to the COL11-12 and COL9-10 lineages, although the precise placement of the OMG1 group remains unresolved.

**Figure 7.**
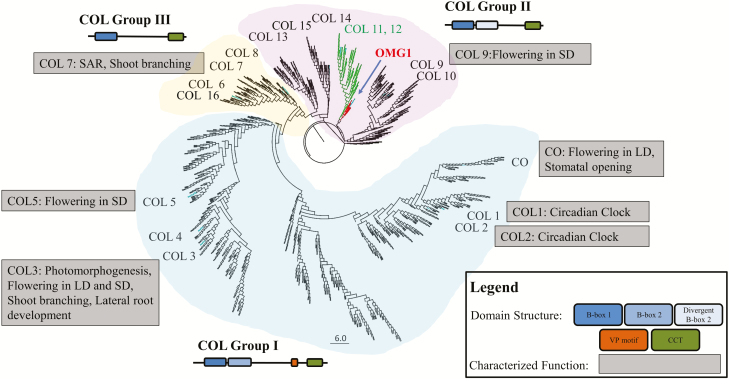
OMG1 is a member of the CO and COL protein family in *Arabidopsis*. The consensus tree derived from full-length protein sequences containing a single CCT domain of all plants found in the non-redundant (NR) database revealed that OMG1 belongs to the CO and COL family of proteins. In *Arabidopsis* (taxa highlighted in cyan), the COL family can be divided into three groups. Group I (shaded in light blue) comprises of CO, COL1-6 which have domain structures consisting of two B-box domains (type I and type II) at the N-terminal and both a VP motif along with a CCT domain at the C-terminal. Genes in group II (shaded in light purple) comprising of COL9-15 and OMG1 have domain structure consisting of a type I B-box domain and a second diverged B-box2 domain along with a C-terminal CCT domain. The OMG1 clade is highlighted in red, whereas its closest homolog COL11-12 is highlighted in green. Group III (shaded in light orange) comprises of COL6-8 and COL16 have domain structures consisting of a single B-box1 domain and a CCT domain. Listed in the grey boxes are the characterized functions of some of the COL members (Gangappa and Botto 2014).

Both the OMG1 and the COL11-12 clades consist of sequences representing both monocots and dicots. In the consensus tree, a single COL11-12-like sequence from the basal flowering plant *Amborella trichopoda* rooted the OMG1 and COL11-12 clades (SH-like aLRT > 0.912), suggesting that the OMG1 lineage arose via a gene-duplication event in flowering plants, prior to the divergence of monocots and dicots (Fig. 7). However, no OMG1-related sequences from Eudicots were observed outside the Brassicaceae family, suggesting that either OMG1 was lost from non-Brassica dicots, or the sequence has diverged so much in these lineages as to be unrecognizable. The different alignment and tree-reconstruction procedures support the early-duplication hypothesis, and make it unlikely that the grouping of monocot and dicot OMG1-related sequences is a phylogenetic artefact.

**Figure 8.**
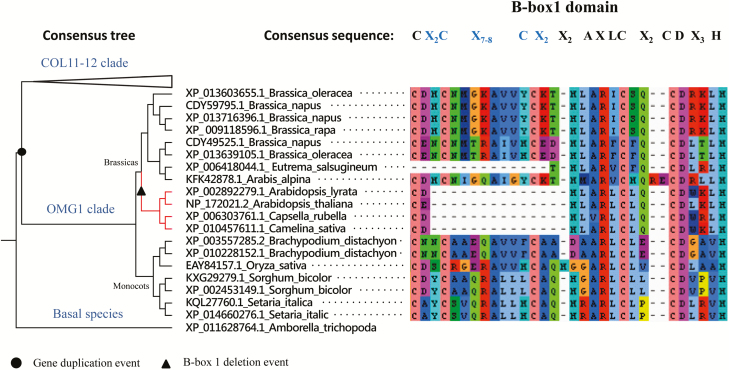
OMG1 resulted from a gene duplication of COL11 and 12 and underwent a subsequent B-box1 deletion event. OMG1 and CONSTANS-Like (COL) 11-12 are sister clades based on the consensus tree described in Fig. 8, suggesting that OMG1 resulted from a gene duplication of COL11-12 (indicated by the circle). Sequences within the OMG1 clade are grouped into *Brassicas* and *monocots*. The *Brassica* sequences highlighted in red, share a similar B-box1 deletion indicated by the triangle and reflected in the adjacent B-box1 domain sequences derived using the AliView software. The B-box1 consensus sequence (Gangappa and Botto 2014) is listed and regions where the deletion occurs are highlighted in blue. The consensus tree depicted here is not drawn to scale.

The high level of sequence divergence in the OMG1 B-box1 domain is primarily due to a 13-amino acid deletion close to the start of the B-box1 consensus sequence CX-------------XAXLCX_2_CDX_3_H (Fig. 7). This B-box1 deletion was observed across several *Brassica* sequences nested within the OMG1 clade, but this deletion was not found in monocot OMG1, suggesting it probably occurred late in the Brassicaceae lineage. Although the OMG1 B-box2 domain is largely conserved (Fig. 8), two highly conserved consensus amino acids are different in OMG1 compared to the consensus sequence CX_2_CX_7-10_CX_7_CX_2_CX_5-12_H. The third cysteine in the consensus sequence is serine in OMG1 (C49S), and the histidine at the end of the consensus sequence is leucine (H73L). Examination of B-box2 domains outside the OMG1 clade suggests that these substitutions are unique to the OMG1 clade **[see****Supporting Information—Fig. S3****]**.

**Figure 9.**
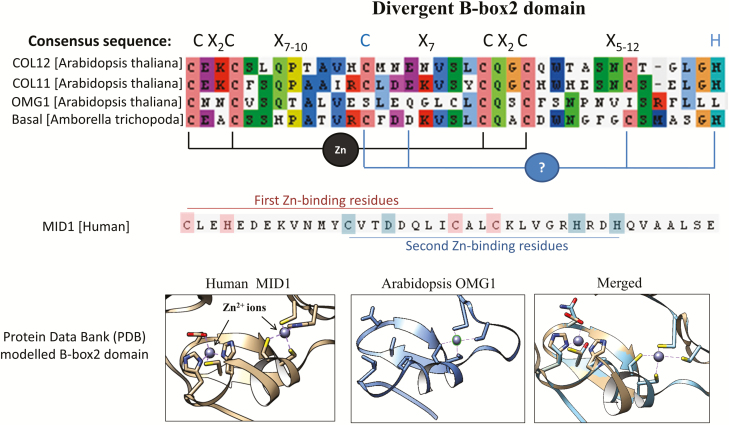
The B-box2 domain of OMG1 is predicted to bind at least one zinc ion. The sequence of B-box2 domain was compared between *Arabidopsis* COL11-12, OMG1 and the basal species *Amborella trichopoda.* The consensus sequence of the B-box2 domain is listed and OMG1 seems to have a relatively conserved B-box2 domain except for the residues highlighted in blue. Residues within the COL sequences that create a zinc-binding interface are indicated by the black lines, whereas residues that might create a second zinc-binding interface are indicated by the blue lines. The human MID1 sequence is also listed and residues that bind to the first zinc ion are highlighted in pink, whereas residues that bind to the second zinc ion are highlighted in blue. OMG1 B-box2 domain was modelled after a well-characterized B-box2 found in the Human MID1 protein located in the Protein Data Bank (PDB). Modelling of the protein was done using the Chimera protein modelling software. In the *Arabidopsis* OMG1 protein, four cysteines are positioned such that they interact with a zinc ion. However, the substitution of amino acids around the second zinc-binding region shows strong evidence for the loss of the second Zn-binding site as modelled in the human MID1 template.

The B-box domain family belongs to a subgroup of zinc-binding proteins stabilized by canonical cysteine-rich zinc-finger motifs (Klug and Schwabe 1995). The inferred structure of Zn-bound OMG1 B-box2 suggests that OMG1 is capable of stably binding at least one zinc ion, via a canonical zinc-finger motif (Fig. 9). In the model, four conserved cysteine residues surrounding the CX_2_C configuration were spatially positioned in a similar fashion as the amino acids surrounding the first Zn-binding site in the human MID1 template, suggesting this first OMG1 Zn-binding site retains a canonical zinc-finger structure.

However, radical substitutions at key Zn-binding sites (C49S, E52Q, C67I and H73L) occurring in the second Zn-binding pocket of OMG1’s B-box2 motif appear to completely disrupt potential Zn-binding (Fig. 9). It remains unclear whether the series of indels and substitutions after the OMG1 B-box2 domain could result in a novel structure that facilitates zinc ion binding or a different functional motif. Furthermore, sequences within the OMG1 clade show similar substitutions at most of these conserved Zn-binding residues, implicating the possibility that functional B-box2 in these proteins require only a single Zn ion **[see****Supporting Information—Fig. S3****]**.

### OMG1 localizes to the nucleus

**Figure 10.**
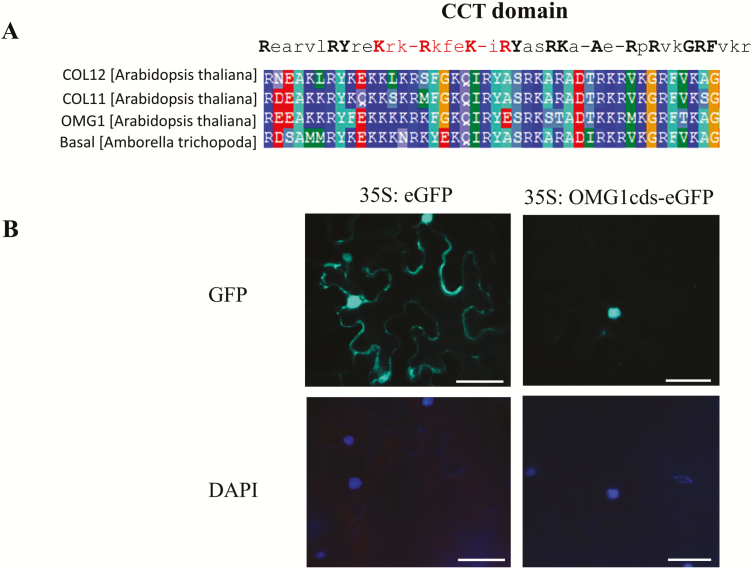
OMG1 has a conserved CCT domain and localizes in the nucleus. (A) The sequence of CCT domain was compared between *Arabidopsis* COL11-12, OMG1 and the basal species *Amborella trichopoda.* OMG1 has a conserved CCT domain (consensus sequence in black) that encodes a nuclear localization signal (highlighted in red). (B) Subcellular localization using leaves of 4-week-old *Nicotiana benthamiana* seedlings that were transiently transformed with *Agrobacterium*-GV3101 carrying the At1g05290cds-eGFP fusion vector or the pCAMBIA1302 (as an overexpressing eGFP control) indicated that OMG1 is localized solely in the nucleus and exhibits no presence elsewhere in the cell. DAPI stains for the nucleus and can be seen to overlap with the eGFP expression. Scale bars: 100 µm.

The transient expression of a 35S::OMG1-eGFP fusion protein in tobacco leaves indicates that OMG1 localizes in the nucleus. The highly conserved CCT domain in OMG1 has been associated with important functions in transcriptional regulation, DNA binding and nuclear localization (Robson *et al.* 2001; Yan *et al.* 2011; Gendron *et al.* 2012). Cells transformed with 35S::eGFP alone displayed a uniform expression of eGFP throughout the leaf pavement cells in the vacuoles, nucleus and membrane. However, cells transformed with 35S::OMG1-eGFP fusion were specifically localized to the nucleus, as indicated by the DAPI nuclei-specific counter stain (Fig. 10).

## Discussion

As sessile organisms, plants have developed sensitive, adaptive strategies to regulate their metabolism in a changing environment (Noctor *et al.* 2007; Schwarzländer and Finkemeier 2012). A strategy to avoid oxidative damage relies on the ability to deploy different antioxidants and ROS-scavenging/detoxifying enzymes to efficiently adjust their redox states. When subjected to the novel environment of spaceflight, plants respond by overexpressing more ROS- or redox-associated genes than comparable plants on the ground (Im *et al.* 2011; Paul *et al.* 2012b, 2013b, 2017; Correll *et al.* 2013; Sugimoto *et al.* 2014). ROS in its various forms and subcellular production sites can elicit distinct and diverse physiological, biochemical and molecular responses (Mittler *et al.* 2004; Gadjev *et al.* 2006; Swanson and Gilroy 2010; Suzuki *et al.* 2012). It is possible that plants drew from diverse ROS response strategies to cope with spaceflight, an environment that is outside their evolutionary experience and therefore potentially stressful. The data presented here suggest that *OMG1* is a putative regulator of the ROS signalling pathway associated with wounding and may play a prominent role in regulating the spaceflight ROS response, especially in the WS ecotype where it is strongly induced by spaceflight.

### Spaceflight induces the differential expression of ROS-producing genes in an ecotype-dependent manner

In general, the differentially expressed genes in spaceflight showed a positive correlation to genes producing ROS (Fig. 1A). Although it is known that ecotype differences can diversify the ROS signatures associated with stress responses on Earth (Kalbina and Strid 2006; Li *et al.* 2006, 2008), these results are the first to demonstrate that different ecotypes use different specific subcellular ROS species to respond to the spaceflight environment (Fig. 1).

Previous morphological differences between Col-0 and WS ecotypes in the spaceflight environment were primarily observed in the roots. Although the overall root length at 11 days on orbit between the two ecotypes showed no significant difference (Paul *et al.* 2017), it was noted that the Col-0 ecotype showed a dramatic lag in root development during the first 48-h post-germination in orbit when compared to the WS ecotype, and that there are also ecotype-specific differences in root growth behaviour (Paul *et al.* 2012a). One difference between the Col-0 and WS ecotypes is the regulation of class III peroxidase genes (Passardi *et al.* 2007). In fact, two genes *PE10* (AT1G49570) and *PE59* (AT5G19890) that were up-regulated in the APEX03-2 Col-0 but remained unchanged in APEX03-2 WS (Fig. 1B) are involved in controlling the root cell elongation process (Markakis *et al.* 2012). The differentially expressed peroxidase genes between the two ecotypes in the APEX03-2 spaceflight transcriptomes (Fig. 1B) reinforce the notion that different ecotypes utilize ROS differently to respond to their environment and suggest that in WS, these genes were already at a level appropriate for spaceflight acclimation and were therefore unchanged. In addition, the observed down-regulation of peroxidases in Col-0 was consonant with observations previously published (Kwon *et al.* 2015). Since peroxidases are necessary for the general oxidoreduction of H_2_O_2_ in general, the down-regulation of peroxidases in spaceflight within Col-0 could contribute to Col-0-specific ROS signatures seen in clusters A and B of the ROSMETER analyses (Fig. 1A).

The current ROSMETER analysis is not intended to define how specific ecotypes utilize peroxidases or regulate specific ROS indices in response to spaceflight. Rather, it is to illustrate that the genetic background of the spaceflight-grown plant had a substantial impact on the specific subcellular ROS species that were used in the physiological response.

### OMG1 transcript is consistently up-regulated in WS ecotype across multiple spaceflight experiments and is induced rapidly upon wounding in both WS and Col-0

*OMG1* is consistently induced in spaceflight in WS but not Col-0. Comparisons of the 5′ upstream region, the coding region and the 3′ UTR of *OMG1* between the two ecotypes showed no differences in the primary sequence. However, in the intergenic region downstream of the *OMG1* stop codon, a point mutation is observed at positions 2197, 3327, and a two-nucleotide deletion is observed at position 3488 **[see****Supporting Information—Fig. S4****]**.

Wassilewskija and Col-0 do regulate *OMG1* differently. *OMG1* showed slight differences in basal expression between Col-0 and WS, and both ecotypes demonstrated immediate up-regulation of *OMG1* after wounding, along with the rapid diminishing of the signal afterwards **[see****Supporting Information—Fig. S5A****]**. However, Col-0 and WS responded very differently in a wounding dosage test, where leaves were subjected to various degrees of damage at the onset of wounding (‘immediate’ time point) **[see****Supporting Information—Fig. S5B****]**. Irrespective of the extent of the wounded surface area of the leaf, Col-0 expressed *OMG1* at a consistent level, whereas WS expressed *OMG1* in a dose-response manner **[see****Supporting Information—Fig. S5C****]**. These results suggest that OMG1 is involved in a rapid wounding response in both Col-0 and WS ecotypes but with different modes of regulation.

The WS and Col-0 ecotypes are genotypically distinct, and their transcriptomic responses to environmental and developmental cues can be dissimilar (e.g. Chen *et al.* 2005; Kusunoki *et al.* 2017). The overall spaceflight transcriptomes of WS and Col-0 are dramatically different (Paul *et al.* 2017). It is hypothesized that even though OMG1 is functional in both ecotypes upon wounding, other gene products or pathways present in Col-0 but not WS could compensate for the ‘need’ for OMG1 in space, or that these genes were already at a level appropriate for spaceflight acclimation and were therefore unchanged in the spaceflight transcriptome. Although OMG1’s role in the physiological adaptation of WS to spaceflight has yet to be discerned, its strong induction suggests that OMG1 contributes to a successful adjustment to that environment. Irrespective of OMG1’s specific role in spaceflight physiology, it is noteworthy that OMG1’s overall metabolic function, which was previously unknown, was only discovered because of its role in spaceflight physiology.

### OMG1 is associated with the regulation of ROS

*OMG1* is associated with ROS regulation. The location of *OMG1* promoter region activity coincided with regions of dynamic ROS activity. *OMG1* specifically regulated the expression of two ROS-associated genes (*GRX480* and *MYB77*). And, loss of OMG1 protein in *omg1* resulted in deviations from WT in two different ROS assays.

The rapid induction of *OMG1* expression immediately after wounding was observed with a promoter-driven GUS reporter qRT-PCR analysis. Rapid induction of transcription (prior to 2-min post-stimulus) has been observed in gravitational and mechanical stimulation of *Arabidopsis* root apical tissues (Kimbrough *et al.* 2004). In addition, wounding *Arabidopsis* leaves produces ROS within minutes at the wound site (Miller *et al.* 2009; Beneloujaephajri *et al.* 2013) and this rapid response is necessary for plants to elicit immediate defence/repair mechanisms against herbivore and pathogen attacks. Wounded plants can transmit rapid RBOHD-dependent ROS signals of speeds up to 8.4 cm min^−1^ to mediate long-distance cell-to-cell communication (Miller *et al.* 2009). However, since no statistical differences were seen in the expression level of *RbohD* in Col-0 WT plants and *omg1* KO plants (Fig. 6D), this suggests that the changes in extracellular or intracellular ROS are not a result of altered expression of *RbohD* in the *omg1* KO line.

Reactive oxygen species molecules are essential signalling molecules in plant growth and development but can also act as toxic stressors. Therefore, there exists an elaborate and highly redundant network of genes that maintain the delicate ROS homeostasis (Gechev *et al.* 2006). Functional OMG1 is necessary for rapid generation of intracellular ROS at the wound site (Fig. 6A and B). However, over time these ROS return to WT levels, presumably facilitated by other genes of redundant function **[see****Supporting Information—Fig. S6****]**.

In a recent review, it has been suggested that altered ROS signalling in response to the spaceflight environment could affect plant growth and development indirectly via changes in hormonal influx within the plants (Schüler *et al.* 2015). OMG1 was found to regulate the expression of ROS-associated genes *GRX480* and *MYB77*. GRX480/GRXC9 is a member of the glutaredoxin (GRX) family of small redox enzymes. GRX480 is a salicylic acid (SA)-responsive gene induced quickly and transiently by an NPR1-independent pathway, which is associated with the ROS-scavenging/antioxidant network (Laporte *et al.* 2012; Zander *et al.* 2012; Herrera-Vásquez *et al.* 2015a; Huang *et al.* 2016). *MYB77* is a member of the R2R3-type transcription factor family that is involved in ROS metabolism through the mitogen-activated protein kinase (MAPK) cascade. The MAPK cascade is also known to play a role in plant development and hormone signalling and includes hormones such as jasmonic acid (JA), SA and ethylene (ET) (Shin and Schachtman 2004; Teige *et al.* 2004; Galvez-Valdivieso *et al.* 2009; Herrera-Vásquez *et al.* 2015b).

OMG1’s role in ROS signalling was established with two frequently used ROS biochemical assays. The DCF-DA assay showed that intracellular ROS production around the wound site was dampened in the *omg1* mutant, whereas flg22-induced extracellular ROS production was 20 % higher than WT in the *omg1* mutant. These conflicting results can be addressed by the different signalling pathways elicited by each stimulus. Responses to mechanical damage can be localized, systemic or both. In any case, the response involves the generation, translocation, perception and transduction of wound signals to activate the expression of wound‐inducible genes. In response to mechanical wounding, the central role for JA signalling is well established. However, many other compounds, such as oligopeptide systemin, oligosaccharides, phytohormones, hydraulic pressure or electrical pulses, have also been proposed to play a role in wound signalling (León *et al.* 2001). Early localized ROS induced by wounding, specifically O_2_•−, H_2_O_2_ and singlet oxygen, have been suggested to originate intracellularly from the photosynthetic electron transport chain in chloroplasts (Morker and Roberts 2011).

On the other hand, flg22, a microbe-associated molecular pattern (MAMP), elicits an early response that includes SA-, JA- and ET-defence signalling pathways. Flg22 also elicits a late response that includes activation of defence-related senescence processes, SA-dependent secretory pathway genes and pathogenesis-related protein 1 (*PR1*) expression (Denoux *et al.* 2008). MAMP-induced ROS have been shown to originate from the apoplast through activation of NADPH oxidases as well as peroxidases (Jwa and Hwang 2017). Although there are crosstalks between signalling pathways induced by mechanical wounding and flg22, there are also unique pathways induced by each stimulus that could attribute to the different results observed. Details of these mechanisms are still being investigated; however, the results infer that within the Brassicaceae family OMG1 does play a role in the ROS signalling pathway.

### Phylogeny and protein architecture of OMG1

Prior to this study, members of the *Arabidopsis* CO and COL family were thought to only function in photoperiod signalling that involves flowering, circadian rhythms, stomata opening and root-shoot branching (Gangappa and Botto 2014). Evidence presented here suggests that members of this family play a role in ROS signalling. This, in turn, could aid in the functional characterization of many of the other uncharacterized members such as COL11 and COL12. We suggest that divergent function of OMG1 can be attributed to the evolved B-box1 and B-box2 domains. This is in line with reports on the rapid evolution of COL genes within the Brassicaceae, in which the increased evolutionary rates were observed not only in the middle region of the protein but also in the conserved zinc-finger and basic CCT regions (Lagercrantz and Axelsson 2000).

## Conclusion

OMG1 is a CCT domain transcriptional regulator of ROS homeostasis, one that is important to the physiological adaptation of *Arabidopsis* ecotype WS to spaceflight. While investigations are underway to determine the detailed mechanisms of how OMG1 regulates the ROS homeostasis in wounding, and its specific role in spaceflight, the results presented here demonstrate several important concepts for general consideration. First, spaceflight experiments are useful platforms to discover novel gene functions and provide fundamental insights into terrestrial plant biology. Second, the response to spaceflight is genotype-specific and this understanding should be taken into consideration when defining the overall plant spaceflight response. WS and Col-0 differ in induction of OMG1 in spaceflight. Third, the maintenance of ROS homeostasis plays an important role as plants physiologically adapt to spaceflight. And, last, the CONSTANS-Like family contains members that function well beyond the clock and timing roles typically associated with CCT domains, including ROS signalling.

## Sources of Funding

This work was supported by NASA - Space Life and Physical Sciences (NNX09AL96G, NNX07AH270, NNX12AN69G and NNX14AT24G awarded to R.J.F. and A.-L.P.).

## Contributions by the Authors

N.J.S. designed and conducted the ROS and OMG1-related experiments, analysed the transcriptome data, and wrote the first drafts of the manuscript. B.K. made substantial contributions to the phylogenetic analyses. A.-L.P. and R.J.F. supervised the work, designed and conducted the spaceflight transcriptome experiments, and revised the manuscript.

## Conflict of Interest

None declared.

## Supporting Information

The following additional information is available in the online version of this article—

**Table S1**. List of all the primer pairs used in experiments presented in this paper.

**Table S2**. List of genes shared between spaceflight, rapid wounding and pollen tube growth transcriptomic data sets.

**Table S3**. List of all unique plant species represented in the phylogenetic analysis.

**Figure S1**. Characterization of the *omg1* homozygous knockout (KO) line (SALK_045742C).

**Figure S2**. Full-length sequence of *Arabidopsis* OMG1 aligned to *Arabidopsis* COL11-12 and the basal species *Amborella trichopoda*.

**Figure S3**. B-box2 domain sequence in all species within the OMG1 clade.

**Figure S4**. Alignment details of OMG1 in Wassilewskija (WS) vs. Columbia (Col-0) cultivar.

**Figure S5**. Characterization of OMG1 expression in wild-type (WT) Wassilewskija (WS) and WT Columbia (Col-0) with qRT-PCR analysis upon wounding experiments.

**Figure S6**. Time course of the 5-(and 6)-carboxy-2′,7′-dichloro dihydrofluorescein diacetate (DCF-DA) fluorescent reactive oxygen species (ROS) assay described in Fig. 5.

Supplementary Material
