# Supplementary material for: A member of the CONSTANS-Like protein family is a putative regulator of reactive oxygen species homeostasis and spaceflight physiological adaptation
Source: AoB Plants. 2018 Dec 15;11(1):ply075. doi: 10.1093/aobpla/ply075 (PMC6348315; doi:10.1093/aobpla/ply075)
Supplement: Supplementary Material [file ply075_suppl_supplementary_material.pdf]

Table S1. List of all primer pairs used in experiments described in the paper.

Primer Set	Description	Forward Primer	Reverse Primer	Probe / Additional Primer
A	OMG1 5' flanking region	5’-CCGGATCCACTTTTCTACAACCCGAATGG-3’	5’-GGCCATGGTTTTTACACTTAATTTAAAAAC-3’	
B	GUSA SYBR RT-qPCR	5’-GTTCTGCGACGCTCACACCGATACC-3’	5’-TCACCGAAGTTCATGCCAGTCCAG-3’	
C	UBQ11 SYBR RT-qPCR	5’-AGCAACTTGAGGACGGCAGA-3’	5’-GTGATGGTCTTTCCGGTCAAA-3’	
D	OMG1 Taqman RT-qPCR	5’-CTCACGGAGCTAATCGATGAATT-3’	5’-CGATGACATACCAGATGCTGAAG-3’	5’-6FAM-CAGAGACCACGCCGACCAATACTTTACCA-TAMRA-3’
E	UBQ11 Taqman RT-qPCR	5’-AACTTGAGGACGGCAGAACTTT-3’	5’-GTGATGGTCTTTCCGGTCAAA-3’	5’-VIC-CAGAAGGAGTCTACGCTTCATTTGGTCTTGC-TAMRA-3’
F	SALK_045742C primers	LP: 5’-AGACCGCTCTTGTAGAATCCC-3’	RP: 5’-TAATTTGTTTCCCAAACCTGC-3’	LBb1.3: 5’ ATTTTGCCGATTTCGGAAC-3’
G	GRX480 SYBR RT-qPCR	5’-GCTGCTTCTTGGACTTGGAG-3’	5’- TAAACCGCCGGTAACTTCAC-3’	
H	PUMP5 SYBR RT-qPCR	5’- AGCGTTGCGAGTAATCCTGT-3’	5’- TCAACCGCTCCTTTATACGG-3’	
I	OMG1 cds for fusion	5’-AACTGCAGATGGGATCACCATTGTGCGAGC-3’	5’-CCACTAGTTGTTTGTATTTTCCCTTTGTTAA-3’	
J	sGFP cds for fusion	5’-ATACTAGTAAGGGCGAGGAGCTGTT-3’	5’-CTGGTCACCTTACTTGTACAGCTCG-3’	
K	OMG1-sGFP fusion	5’-AACCATGGATGGGATCACCATTGTGCGAGC-3’	5’-CTGGTCACCTTACTTGTACAGCTCG-3’	
L	XT1 SYBR RT-qPCR	5’-TGGGATGAGCAGAGACGTGA-3’	5’-GCGCCACGAAATTAGGGAAG-3’	
M	TCH4 SYBR RT-qPCR	5’-GGCACTCTGTTTCCCAAGAACA-3’	5’-CTCGTTGCCCAATCATCAGC-3’	
N	MYB77 SYBR RT-qPCR	5’-GCGTTGATGTTTCCGAGATT-3’	5’-TTTCCGCCATGTAACTCCTC-3’	
O	TCH2 SYBR RT-qPCR	5’-AATCGGAGGAGGAGGTAACAA-3’	5’-CCGAGATCCTTCCATTACCA-3’	
P	PBP1 SYBR RT-qPCR	5’-TCCCAACAATGGCAGGAAA-3’	5’-GCTCGAACCCTTTGCAAATCT-3’	
Q	ACS6 SYBR RT-qPCR	5’-TGGGTCTGCCTGGTTTAAGAG-3’	5’-TGCGATCTGAACCACCCTGT-3’	
R	RBOHD SYBR RT-qPCR	5’-CTGGACACGTAAGCTCAGGA-3’	5’-GCCGAGACCTACGAGGAGTA-3’	

Table S1. List of all the primer pairs used in experiments presented in this paper. Primer sequences used in experiments presented in this paper are listed in the table.

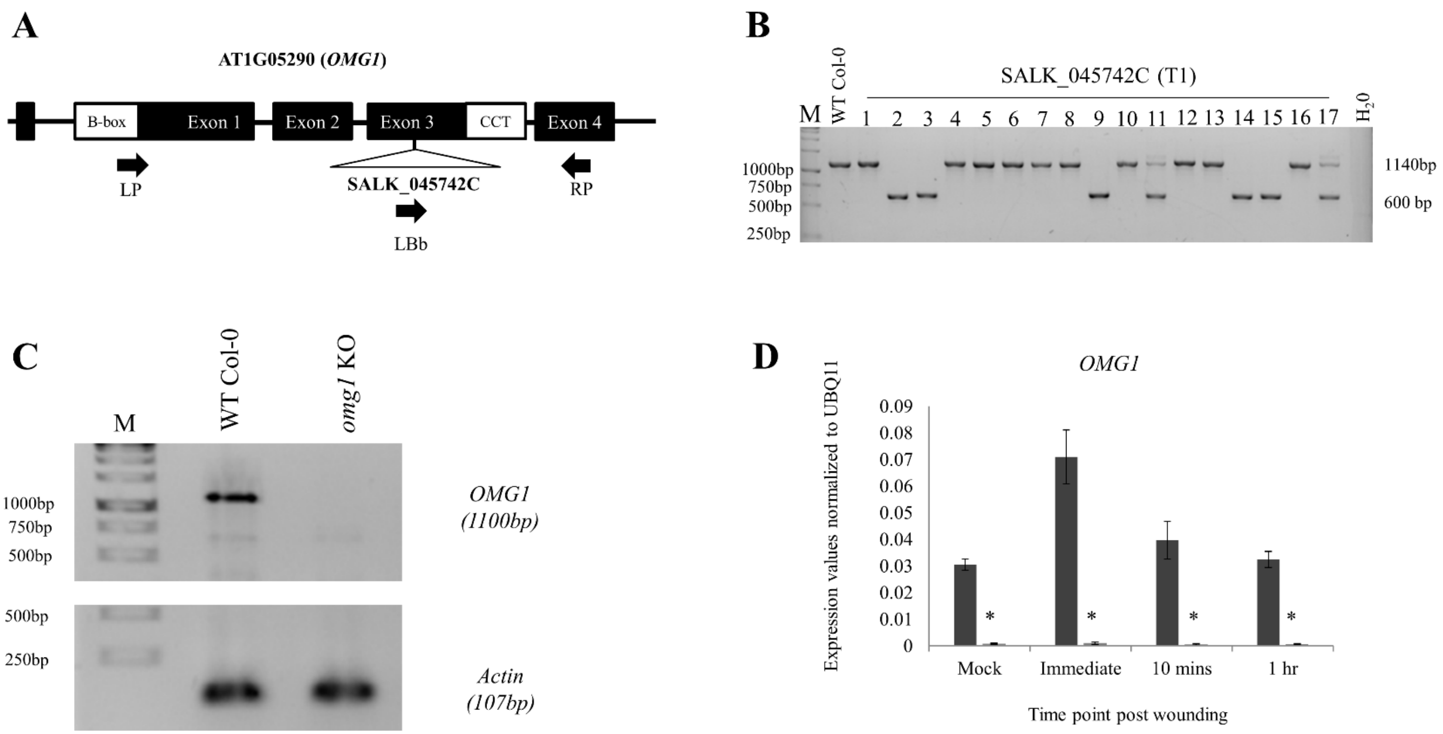

Figure S1. Characterization of the omg1 homozygous KO line (SALK_045742C). A) Shows a schematic diagram of SALK_045742C line T-DNA insertion in exon 3 of OMG1. Black arrows indicate primers derived from the SALKSignal T-DNA primer design web tool (http://signal.salk.edu/tdnaprimers.2.html) used to screen the SALK line. B) The same primers illustrated in Fig. S3A [LP + LBb + RP (Table S1, set F)] were used to screen the SALK_045742C T1 seeds received from the Salk Institute Genomic Analysis Laboratory. PCR gel showed a mixture of wild type (1140bp), Homozygous (600bp) and heterozygous (1140bp +600bp) lines. After selfing the homozygous lines, all T3 progenies were homozygous for the T-DNA insertion. These seeds were then used as the omg1 KO line for downstream analyses. C) RT-PCR gel for the full length OMG1 cDNA in Col-0 WT and omg1 KO lines was done to ensure that the OMG1 transcript is not present in the KO line. Primers used are listed in Table S1, set I. Actin was used as a loading control. M denotes the marker. D) OMG1 transcript abundance was measured after wounding for the indicated time points in both WT-Col and omg1 KO. OMG1 transcripts were not detected in the omg1 KO line.

Table S2. List of genes shared between spaceflight experiments, rapid wounding and pollen tube growth.

	ATG #	Gene name	TAGES roots[1]		30mins post wounding(RtS)[2]		Pollen germination and tube growth[3]		Details[4]
			Pvalue	FC	Pvalue	FC	Pvalue	FC	
1	At1g28480	GRX480	5.98E-03	3.6	1.80E-01	3.6			Regulates protein redox state by detoxifying reactive chemicals
2	At2g22500	PUMP5	3.27E-03	3.2	1.70E-03	3.1	4.00E-05	2.39	Oxidative phosphorylation uncoupler activity
3	At3g62720	XXT1	2.75E-03	2.7	1.60E-03	2.4	2.00E-05	2.58	Enzyme that adds several xylosyl residues on to polysaccharides
4	At5g37770	TCH2	7.94E-03	2.1	6.20E-04	2.5	2.00E-05	4.87	Response to calcium
5	At4g11280	ACS6	5.88E-03	3.2	8.60E-04	2.5	3.00E-05	2.32	Respond to mechanical stimuli
6	At3g50060	MYB77	3.17E-03	3.6	2.30E-02	3.3	8.00E-05	3.93	TF involved in lateral root development and ROS metabolism
7	At5g54490	PBP1	6.52E-04	3.6	1.20E-01	2.8			Response to calcium
8	At5g57560	TCH4	2.80E-03	5.1					Respond to mechanical stimuli

Table S2. List of genes shared between spaceflight, rapid wounding and pollen tube growth transcriptomic datasets. The list was derived by comparing the genes that were differentially expressed in spaceflight1 (Paul et al, 2013) against those shared in rapid wounding2 (Hasegawa et al, 2011; Supplemental Table S2) and in pollen tube germination & growth3 (Wang et al, 2008; Supplemental Table S2). The list of 7 genes with the respective ATG numbers, gene names, and functional details are listed along with the p-values and fold change from the respective experiments. Functional details4 of each gene were analyzed using available resources such as TAIR and PubMed.

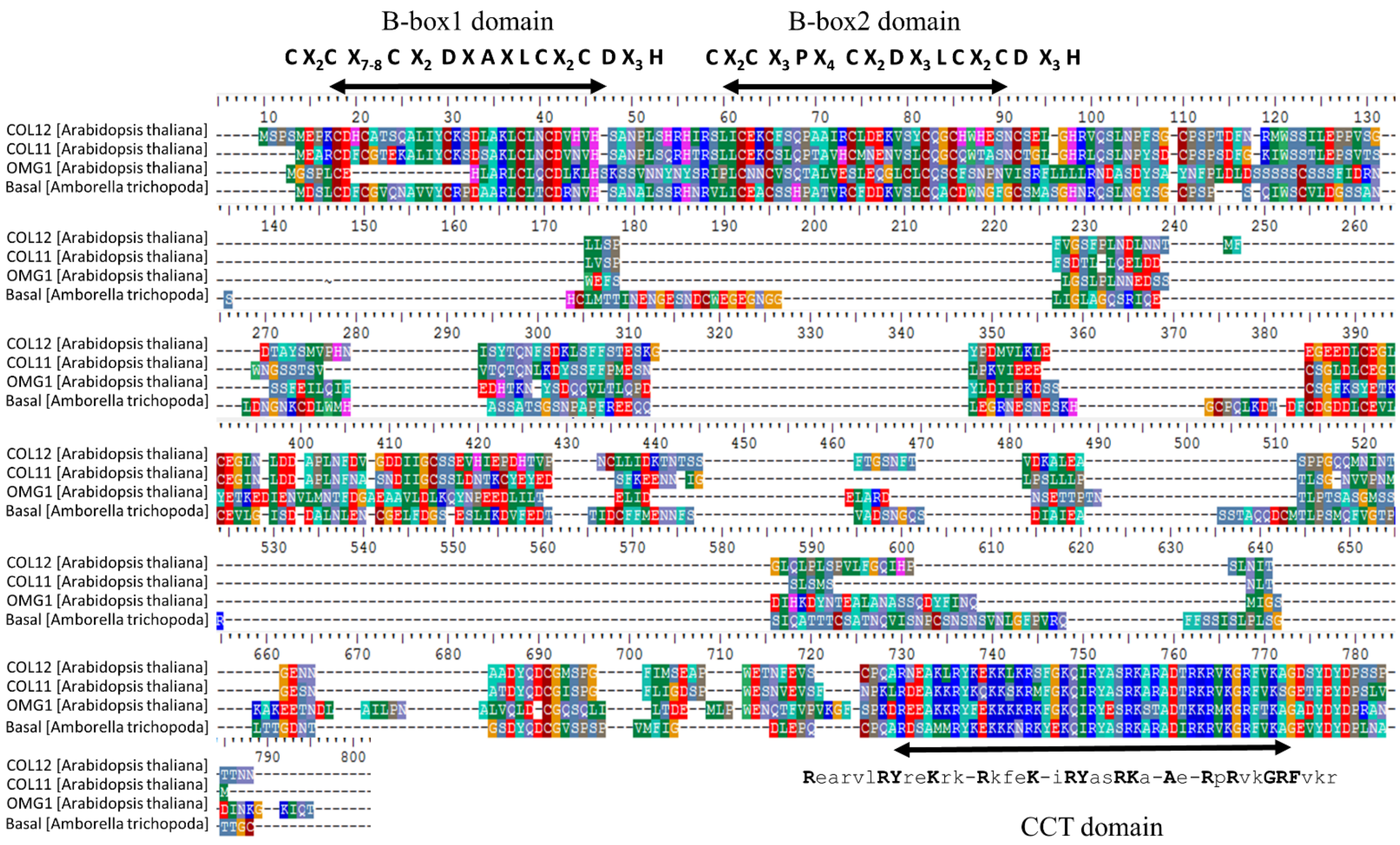

Figure S2. Full length sequence of Arabidopsis OMG1 aligned to Arabidopsis COL11-12 and the basal species *Amborella trichopoda*. Full length sequence of Arabidopsis OMG1 aligned to its closest homolog Arabidopsis COL11-12 and the basal species of that clade *Amborella trichopoda*. The alignment was done using default settings in the BioEdit software. The regions of conserved domains are indicated by the black arrows and the consensus sequences are listed for each domain.

Table S3. List of all unique plant species represented in the phylogenetic analysis.

No	Unique species	No	Unique species	No	Unique species
1	Aegilops_tauschii	56	Festuca_pratensis	111	Oryza_nivara
2	Agapanthus_praecox	57	Fragaria_vesca	112	Oryza_officinalis
3	Allium_cepa	58	Fragaria_x	113	Oryza_rufipogon
4	Amborella_trichopoda	59	Galdieria_sulphuraria	114	Oryza_sativa
5	Ananas_comosus	60	Genlisea_aurea	115	Ostreococcus_lucimarinus
6	Annona_squamosa	61	Gentiana_triflora	116	Ostreococcus_tauri
7	Aquilegia_formosa	62	Glycine_max	117	Oxybasis_rubra
8	Arabidopsis_halleri	63	Glycine_soja	118	Paeonia_suffruticosa
9	Arabidopsis_kamchatica	64	Gonium_pectorale	119	Petunia_x
10	Arabidopsis_lyrata	65	Gossypium_arboreum	120	Phalaenopsis_hybrid
11	Arabidopsis_thaliana	66	Gossypium_barbadense	121	Phaseolus_vulgaris
12	Arabis_alpina	67	Gossypium_darwinii	122	Phoenix_dactylifera
13	Arachis_duranensis	68	Gossypium_herbaceum	123	Phyllostachys_edulis
14	Arachis_hypogaea	69	Gossypium_hirsutum	124	Physcomitrella_patens
15	Arachis_ipaensis	70	Gossypium_mustelinum	125	Picea_abies
16	Beta_vulgaris	71	Gossypium_raimondii	126	Picea_sitchensis
17	Betula_luminifera	72	Gossypium_tomentosum	127	Pinus_pinaster
18	Boehmeria_nivea	73	Helianthus_annuus	128	Pinus_radiata
19	Brachypodium_distachyon	74	Hordeum_vulgare	129	Pinus_sylvestris
20	Brassica_juncea	75	Ipomoea_nil	130	Pisum_sativum
21	Brassica_napus	76	Jatropha_curcas	131	Populus_balsamifera
22	Brassica_nigra	77	Klebsormidium_flaccidum	132	Populus_deltoides
23	Brassica_oleracea	78	Lagerstroemia_indica	133	Populus_euphratica
24	Brassica_rapa	79	Larix_kaempferi	134	Populus_tomentosa
25	Cajanus_cajan	80	Lemna_aequinoctialis	135	Populus_trichocarpa
26	Camelina_sativa	81	Lemna_gibba	136	Primula_vulgaris
27	Capsella_rubella	82	Lilium_hybrid	137	Prunus_mume
28	Capsicum_annuum	83	Litchi_chinensis	138	Prunus_persica
29	Chondrus_crispus	84	Lolium_perenne	139	Pyrus_x
30	Chrysanthemum_seticuspe	85	Lolium_temulentum	140	Raphanus_sativus
31	Chrysanthemum_x	86	Magnolia_virginiana	141	Ricinus_communis
32	Cicer_arietinum	87	Malus_domestica	142	Secale_cereale
33	Citrus_clementina	88	Mangifera_indica	143	Selaginella_moellendorffii
34	Citrus_sinensis	89	Manihot_esculenta	144	Sesamum_indicum
35	Coccomyxa_subellipsoidea	90	Marchantia_polymorpha	145	Setaria_italica
36	Coffea_arabica	91	Medicago_sativa	146	Sinapis_alba
37	Coffea_canephora	92	Medicago_truncatula	147	Solanum_lycopersicum
38	Cucumis_melo	93	Miscanthus_floridulus	148	Solanum_pennellii
39	Cucumis_sativus	94	Miscanthus_sacchariflorus	149	Solanum_tuberosum
40	Cymbidium_ensifolium	95	Miscanthus_sinensis	150	Sorghum_bicolor
41	Cymbidium_goeringii	96	Morus_notabilis	151	Spinacia_oleracea
42	Cymbidium_sinense	97	Musa_AAB	152	Tarenaya_hassleriana
43	Cynara_cardunculus	98	Musa_acuminata	153	Tectona_grandis
44	Daucus_carota	99	Nelumbo_nucifera	154	Theobroma_cacao
45	Dendrobium_loddigesii	100	Nicotiana_sylvestris	155	Thespesia_populneoides
46	Dendrocalamus_xishuangbannaensis	101	Nicotiana_tabacum	156	Trifolium_subterraneum
47	Dimocarpus_longan	102	Nicotiana_tomentosiformis	157	Triticum_aestivum
48	Dorcoceras_hygrometricum	103	Olea_europaea	158	Triticum_urartu
49	Elaeis_guineensis	104	Oryza_australiensis	159	Vigna_angularis
50	Erycina_pusilla	105	Oryza_barthii	160	Vigna_radiata
51	Erythranthe_guttata	106	Oryza_brachyantha	161	Vitis_vinifera

No	Unique species	No	Unique species	No	Unique species
52	Eucalyptus_grandis	107	Oryza_glumipatula	162	Volvox_carteri
53	Eutrema_salsugineum	108	Oryza_granulata	163	Zea_mays
54	Fagus_sylvatica	109	Oryza_longistaminata	164	Ziziphus_jujuba
55	Festuca_arundinacea	110	Oryza_meridionalis	165	Zostera_marina

Table S3. List of all unique plant species represented in the phylogenetic analysis. All the unique plant species represented in the 1547 protein sequences used to generate the phylogenetic tree are listed in this table.

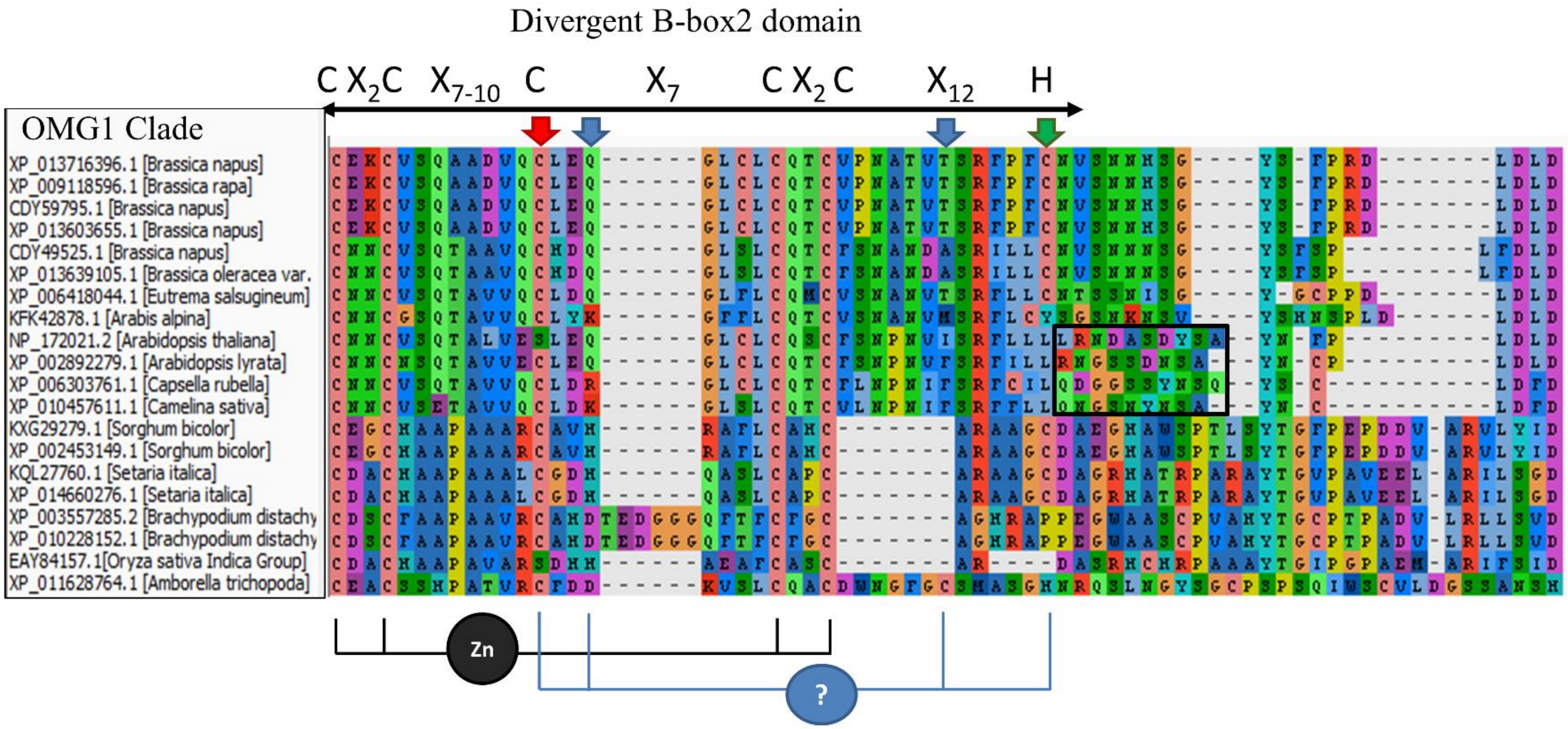

Figure S3. B-box2 domain sequence in all species within the OMG1 clade. The alignment was done with the Aliview software. The sequences highlighted within the black box indicates additional mutations after the B-box2 domain in the same sequences that also harbors the B-box1 deletion (Fig. 8). Residues within the sequences that create the first zinc binding interface are indicated by the black lines, whereas residues that might create a second zinc binding interface with higher uncertainty are indicated by the blue lines. Interestingly, in the consensus sequence listed above, all the residues involved in the second zinc binding interface are not conserved within the OMG1 clade (multicolored arrows). The red arrows indicated the sequences carrying the cysteine to serine substitution. The green arrows indicate the histidine to cysteine substitution. The blue arrows indicate other substitutions from the basal species *Amborella trichopoda*.

```
              T  F  L  Q  P  E  W  F  G  S  V  W  F  N  *  Y  Y  N  S  K
WS     1      ACTTTTCTACAACCCGAATGGTTCGGGTCGGTTTGGTTTAACTAATATTATAACTCTAAA  60
              ||||||||||||||||||||||||||||||||||||||||||||||||||||||||||||
Col-0  1      acttttctacaacccgaatggttcgggtcggtttggtttaactaatattataactctaaa  60
              T  F  L  Q  P  E  W  F  G  S  V  W  F  N  *  Y  Y  N  S  K

              F  *  S  H  A  *  Y  H  I  *  Y  T  I  I  *  Y  K  F  T  N
WS     61     TTCTAAAGTCATGCTTAATATCATATTTAATATACAATTATTTAGTATAAGTTTACTAAT  120
              ||||||||||||||||||||||||||||||||||||||||||||||||||||||||||||
Col-0  61     ttctaaagtcatgcttaatatcatatttaatatacaattatttagtataagtttactaat  120
              F  *  S  H  A  *  Y  H  I  *  Y  T  I  I  *  Y  K  F  T  N

              S  S  N  L  K  I  F  N  S  F  N  N  *  F  *  A  *  M  R  A
WS     121    TCTTCTAATCTCAAAATATTTAATTCGTTTAATAATTAATTCTAAGCTTAGATGAGGGCC  180
              ||||||||||||||||||||||||||||||||||||||||||||||||||||||| ||||
Col-0  121    tcttctaatctcaaaatatttaattcgtttaataattaattctaagcttagatgaaggcc  180
              S  S  N  L  K  I  F  N  S  F  N  N  *  F  *  A  *  M  K  A

              T  L  *  W  R  G  N  D  L  V  T  V  K  F  H  V  G  N  I  I
WS     181    ACTCTCTGATGGCGTGGCAATGATTTAGTCACAGTAAAATTTCATGTGGGAAATATTATA  240
              ||||||||||||||||||||||||||||||||||||||||||||||||||||||||||||
Col-0  181    actctctgatggcgtggcaatgatttagtcacagtaaaatttcatgtgggaaatattata  240
              T  L  *  W  R  G  N  D  L  V  T  V  K  F  H  V  G  N  I  I

              N  C  E  F  W  *  N  F  F  N  N  V  H  A  I  C  K  G  N  I
WS     241    AACTGTGAATTTTGGTGAAATTTCTTCAACAATGTTCATGCAATCTGCAAGGGAAATATA  300
              ||||||||||||||||||||||||||||||||||||||||||||||||||||||||||||
Col-0  241    aactgtgaattttggtgaaatttcttcaacaatgttcatgcaatctgcaagggaaatata  300
              N  C  E  F  W  *  N  F  F  N  N  V  H  A  I  C  K  G  N  I

              T  D  G  Y  R  *  K  S  R  I  K  R  Y  N  S  E  I  Q  N  C
WS     301    ACAGATGGATATAGATGAAAATCAAGGATAAAACGTTATAACAGTGAGATTCAAAACTGT  360
              ||||||||||||||||||||||||||||||||||||||||||||||||||||||||||||
Col-0  301    acagatggatatagatgaaaatcaaggataaaacgttataacagtgagattcaaaactgt  360
              T  D  G  Y  R  *  K  S  R  I  K  R  Y  N  S  E  I  Q  N  C
```

```
             *  I  I  F  R  L  E  R  L  R  T  F  *  P  I  L  I  *  I  L
WS     361   TAAATTATATTTAGACTCGAAAGGTTAAGAACTTTCTAACCAATCTTAATCTAAATTCTC  420
             ||||||||||||||||||||||||||||||||||||||||||||||||||||||||||||
Col-0  361   taaattatatttagactcgaaaggttaagaactttctaaccaatcttaatctaaattctc  420
             *  I  I  F  R  L  E  R  L  R  T  F  *  P  I  L  I  *  I  L

             C  Y  I  F  F  L  F  *  I  I  N  L  K  I  R  N  F  R  *  L
WS     421   TGTTATATTTTTTTCTTATTCTAAATTATAAACTTGAAAATTCGAAATTTTCGCTGATTG  480
             ||||||||||||||||||||||||||||||||||||||||||||||||||||||||||||
Col-0  421   tgttatatttttttcttattctaaattataaacttgaaaattcgaaattttcgctgattg  480
             C  Y  I  F  F  L  F  *  I  I  N  L  K  I  R  N  F  R  *  L

             I  *  F  F  R  H  I  S  N  K  A  V  F  N  G  F  V  Q  K  V
WS     481   ATTTGATTTTTTCGTCATATTTCTAACAAAGCTGTCTTTAACGGTTTCGTCCAAAAAGTT  540
             ||||||||||||||||||||||||||||||||||||||||||||||||||||||||||||
Col-0  481   atttgatttttcgtcatatttctaacaaagctgtctttaacggtttcgtccaaaaagtt  540
             I  *  F  F  R  H  I  S  N  K  A  V  F  N  G  F  V  Q  K  V

             S  Y  Y  L  P  I  C  H  P  K  N  P  I  K  Q  T  E  S  D  Q
WS     541   TCCTACTATTTACCAATCTGCCACCCCAAAAACCCTATCAAGCAAACGGAATCCGATCAA  600
             ||||||||||||||||||||||||||||||||||||||||||||||||||||||||||||
Col-0  541   tcctactatttaccaatctgccaccccaaaaaccctatcaagcaaacggaatccgatcaa  600
             S  Y  Y  L  P  I  C  H  P  K  N  P  I  K  Q  T  E  S  D  Q

             Y  A  F  I  N  S  *  H  S  I  D  I  Y  H  V  *  K  V  T  L
WS     601   TATGCTTTTATTAATTCATAACACAGCATCGATATTTACCACGTGTAAAAAGTAACTCTT  660
             ||||||||||||||||||||||||||||||||||||||||||||||||||||||||||||
Col-0  601   tatgcttttattaattcataacacagcatcgatatttaccacgtgtaaaaagtaactctt  660
             Y  A  F  I  N  S  *  H  S  I  D  I  Y  H  V  *  K  V  T  L

             N  *  Y  K  R  S  T  S  L  R  S  H  I  Y  N  A  F  L  S  F
WS     661   AATTAGTATAAAAGAAGCACAAGCTTGAGATCTCATATCTACAATGCCTTTCTTTCTTTC  720
             ||||||||||||||||||||||||||||||||||||||||||||||||||||||||||||
Col-0  661   aattagtataaaagaagcacaagcttgagatctcatatctacaatgcctttctttctttc  720
             N  *  Y  K  R  S  T  S  L  R  S  H  I  Y  N  A  F  L  S  F
```

```
              K  S  I  D  L  K  F  Y  T  V  R  I  F  F  F  S  F  F  F  X
WS     721    AAATCTATAGATCTCAAATTTTACACTGTTCGTATCTTTTTTTTTTCTTTTTTTTTTTTK  780
              |||||||||||||||||||||||||||||||||||||||||||||||||||||||||||:
Col-0  721    aaatctatagatctcaaattttacactgttcgtatcttttttttttctttttttttttttg  780
              K  S  I  D  L  K  F  Y  T  V  R  I  F  F  F  S  F  F  F  L

              F  L  N  *  V  *  K  M  G  S  P  L  C  E  H  L  A  R  L  C
WS     781    TTTTTAAATTAAGTGTAAAAAATGGGATCACCATTGTGCGAGCATTTGGCTAGGCTTTGT  840
              ||||||||||||||||||||||||||||||||||||||||||||||||||||||||||||
Col-0  781    tttttaaattaagtgtaaaaaATGGGATCACCATTGTGCGAGCATTTGGCTAGGCTTTGT  840
              F  L  N  *  V  *  K  M  G  S  P  L  C  E  H  L  A  R  L  C

              L  Q  C  D  L  K  L  H  S  K  S  S  V  N  N  Y  N  Y  S  R
WS     841    CTTCAATGTGATTTGAAGTTACACTCTAAGAGTAGTGTAAACAATTACAACTATTCGCGG  900
              ||||||||||||||||||||||||||||||||||||||||||||||||||||||||||||
Col-0  841    CTTCAATGTGATTTGAAGTTACACTCTAAGAGTAGTGTAAACAATTACAACTATTCGCGG  900
              L  Q  C  D  L  K  L  H  S  K  S  S  V  N  N  Y  N  Y  S  R

              I  P  L  C  N  N  C  V  S  Q  T  A  L  V  E  S  L  E  Q  G
WS     901    ATTCCGTTGTGTAATAATTGCGTTTCGCAGACCGCTCTTGTAGAATCCCTTGAACAAGGG  960
              ||||||||||||||||||||||||||||||||||||||||||||||||||||||||||||
Col-0  901    ATTCCGTTGTGTAATAATTGCGTTTCGCAGACCGCTCTTGTAGAATCCCTTGAACAAGGG  960
              I  P  L  C  N  N  C  V  S  Q  T  A  L  V  E  S  L  E  Q  G

              L  C  L  C  Q  S  C  F  S  N  P  N  V  I  S  R  F  L  L  L
WS     961    TTGTGCCTATGCCAATCATGCTTTTCAAATCCCAATGTAATCTCGCGTTTTCTTCTTCTT  1020
              ||||||||||||||||||||||||||||||||||||||||||||||||||||||||||||
Col-0  961    TTGTGCCTATGCCAATCATGCTTTTCAAATCCCAATGTAATCTCGCGTTTTCTTCTTCTT  1020
              L  C  L  C  Q  S  C  F  S  N  P  N  V  I  S  R  F  L  L  L

              L  R  N  D  A  S  D  Y  S  A  Y  N  F  P  L  D  L  D  S  S
WS     1021   CTTAGAAACGATGCTAGTGATTATTCGGCATATAATTTTCCACTGGATCTTGATTCCTCT  1080
              ||||||||||||||||||||||||||||||||||||||||||||||||||||||||||||
Col-0  1021   CTTAGAAACGATGCTAGTGATTATTCGGCATATAATTTTCCACTGGATCTTGATTCCTCT  1080
              L  R  N  D  A  S  D  Y  S  A  Y  N  F  P  L  D  L  D  S  S
```

```
                S  S  S  C  S  S  S  F  I  D  R  N  W  E  F  S  L  G  S  L
WS      1081   TCTTCGTCTTGTTCATCTTCTTTTATCGATCGCAATTGGGAATTTTCCCTTGGTTCCTTA  1140
               ||||||||||||||||||||||||||||||||||||||||||||||||||||||||||||
Col-0   1081   TCTTCGTCTTGTTCATCTTCTTTTATCGATCGCAATTGGGAATTTTCCCTTGGTTCCTTA  1140
                S  S  S  C  S  S  S  F  I  D  R  N  W  E  F  S  L  G  S  L

                P  L  N  N  E  D  S  S  S  S  F  E  I  L  Q  I  F  E  D  H
WS      1141   CCTCTCAATAATGAGGACTCATCATCATCTTTTGAAATCCTTCAAATATTTGAAGATCAT  1200
               ||||||||||||||||||||||||||||||||||||||||||||||||||||||||||||
Col-0   1141   CCTCTCAATAATGAGGACTCATCATCATCTTTTGAAATCCTTCAAATATTTGAAGATCAT  1200
                P  L  N  N  E  D  S  S  S  S  F  E  I  L  Q  I  F  E  D  H

                T  K  N  Y  S  D  Q  Q  V  L  T  L  Q  P  D  Y  L  D  I  I
WS      1201   ACAAAGAATTATAGTGATCAACAAGTGTTGACGCTTCAGCCCGATTATTTGGACATAATT  1260
               ||||||||||||||||||||||||||||||||||||||||||||||||||||||||||||
Col-0   1201   ACAAAGAATTATAGTGATCAACAAGTGTTGACGCTTCAGCCCGATTATTTGGACATAATT  1260
                T  K  N  Y  S  D  Q  Q  V  L  T  L  Q  P  D  Y  L  D  I  I

                P  K  V  Y  T  Y  L  F  L  F  C  *  R  G  V  L  F  I  V  M
WS      1261   CCTAAGGTATATACTTATTTATTTTTATTTTGTTAAAGAGGGGTTTTGTTCATTGTTATG  1320
               ||||||||||||||||||||||||||||||||||||||||||||||||||||||||||||
Col-0   1261   CCTAAGgtatatacttatttatttttattttgttaaagaggggttttgttcattgttatg  1320
                P  K  V  Y  T  Y  L  F  L  F  C  *  R  G  V  L  F  I  V  M

                R  Y  I  C  L  L  N  S  F  H  C  F  L  C  V  T  F  T  Q  D
WS      1321   AGATATATATGTTTGCTTAACTCGTTCCATTGTTTCCTGTGTGTCACTTTTACGCAGGAT  1380
               ||||||||||||||||||||||||||||||||||||||||||||||||||||||||||||
Col-0   1321   agatatatatgtttgcttaactcgttccattgtttcctgtgtgtcacttttacgcagGAT  1380
                R  Y  I  C  L  L  N  S  F  H  C  F  L  C  V  T  F  T  Q  D

                S  S  C  S  G  F  K  S  Y  E  T  K  E  D  I  E  N  V  L  M
WS      1381   AGTTCATGCTCAGGCTTCAAAAGCTACGAAACTAAAGAAGATATAGAGAATGTTTTGATG  1440
               ||||||||||||||||||||||||||||||||||||||||||||||||||||||||||||
Col-0   1381   AGTTCATGCTCAGGCTTCAAAAGCTACGAAACTAAAGAAGATATAGAGAATGTTTTGATG  1440
                S  S  C  S  G  F  K  S  Y  E  T  K  E  D  I  E  N  V  L  M
```

```
                 N  T  F  D  G  A  E  A  A  V  L  D  L  K  Q  Y  N  P  E  E
WS      1441   AACACTTTTGATGGCGCCGAGGCAGCAGTATTAGACCTAAAACAATATAACCCCGAGGAA  1500
               ||||||||||||||||||||||||||||||||||||||||||||||||||||||||||||
Col-0   1441   AACACTTTTGATGGCGCCGAGGCAGCAGTATTAGACCTAAAACAATATAACCCCGAGGAA  1500
                 N  T  F  D  G  A  E  A  A  V  L  D  L  K  Q  Y  N  P  E  E

                 D  L  I  L  T  E  L  I  D  E  L  A  R  D  N  S  E  T  T  P
WS      1501   GATTTGATCCTCACGGAGCTAATCGATGAATTAGCAAGAGACAATTCAGAGACCACGCCG  1560
               ||||||||||||||||||||||||||||||||||||||||||||||||||||||||||||
Col-0   1501   GATTTGATCCTCACGGAGCTAATCGATGAATTAGCAAGAGACAATTCAGAGACCACGCCG  1560
                 D  L  I  L  T  E  L  I  D  E  L  A  R  D  N  S  E  T  T  P

                 T  N  V  N  I  S  F  N  S  V  A  Y  *  L  S  L  I  M  V  D
WS      1561   ACCAATGTAAACATCAGCTTTAATTCTGTCGCATATTAATTATCTTTAATTATGGTTGAC  1620
               ||||||||||||||||||||||||||||||||||||||||||||||||||||||||||||
Col-0   1561   ACCAATgtaaacatcagctttaattctgtcgcatattaattatctttaattatggttgac  1620
                 T  N  V  N  I  S  F  N  S  V  A  Y  *  L  S  L  I  M  V  D

                 L  F  I  Y  F  C  F  I  V  *  *  T  L  P  T  S  A  S  G  M
WS      1621   TTATTTATATATTTTTGTTTCATTGTGTAATAGACTTTACCAACTTCAGCATCTGGTATG  1680
               ||||||||||||||||||||||||||||||||||||||||||||||||||||||||||||
Col-0   1621   ttatttatatatttttgtttcattgtgtaatagACTTTACCAACTTCAGCATCTGGTATG  1680
                 L  F  I  Y  F  C  F  I  V  *  *  T  L  P  T  S  A  S  G  M

                 S  S  D  I  H  K  D  Y  N  T  E  A  L  A  N  A  S  S  Q  D
WS      1681   TCATCGGATATACATAAAGATTACAACACTGAAGCTTTGGCTAATGCAAGTTCTCAAGAT  1740
               ||||||||||||||||||||||||||||||||||||||||||||||||||||||||||||
Col-0   1681   TCATCGGATATACATAAAGATTACAACACTGAAGCTTTGGCTAATGCAAGTTCTCAAGAT  1740
                 S  S  D  I  H  K  D  Y  N  T  E  A  L  A  N  A  S  S  Q  D

                 Y  F  I  N  Q  M  I  G  S  K  A  K  E  E  T  N  D  L  A  I
WS      1741   TATTTTATAAACCAAATGATTGGCTCAAAAGCAAAGGAAGAGACCAATGATCTTGCAATA  1800
               ||||||||||||||||||||||||||||||||||||||||||||||||||||||||||||
Col-0   1741   TATTTTATAAACCAAATGATTGGCTCAAAAGCAAAGGAAGAGACCAATGATCTTGCAATA  1800
                 Y  F  I  N  Q  M  I  G  S  K  A  K  E  E  T  N  D  L  A  I
```

```
               L  P  N  A  L  V  Q  L  D  C  G  Q  S  Q  L  I  L  T  D  E
WS     1801  CTCCCTAACGCTCTTGTTCAACTTGACTGTGGACAATCGCAGTTGATACTTACCGATGAG  1860
             ||||||||||||||||||||||||||||||||||||||||||||||||||||||||||||
Col-0  1801  CTCCCTAACGCTCTTGTTCAACTTGACTGTGGACAATCGCAGTTGATACTTACCGATGAG  1860
               L  P  N  A  L  V  Q  L  D  C  G  Q  S  Q  L  I  L  T  D  E

               M  L  P  W  E  N  Q  T  F  V  P  V  K  G  F  S  P  K  D  R
WS     1861  ATGTTACCATGGGAGAATCAAACATTTGTACCTGTTAAGGGATTTAGTCCGAAAGATCGA  1920
             ||||||||||||||||||||||||||||||||||||||||||||||||||||||||||||
Col-0  1861  ATGTTACCATGGGAGAATCAAACATTTGTACCTGTTAAGGGATTTAGTCCGAAAGATCGA  1920
               M  L  P  W  E  N  Q  T  F  V  P  V  K  G  F  S  P  K  D  R

               E  E  A  K  K  R  Y  F  E  K  K  K  K  R  K  *  V  L  H  L
WS     1921  GAAGAGGCCAAGAAAAGATATTTCGAAAAGAAGAAGAAACGCAAGTAGGTATTACACCTC  1980
             ||||||||||||||||||||||||||||||||||||||||||||||||||||||||||||
Col-0  1921  GAAGAGGCCAAGAAAAGATATTTCGAAAAGAAGAAGAAACGCAAgtaggtattacacctc  1980
               E  E  A  K  K  R  Y  F  E  K  K  K  K  R  K  *  V  L  H  L

               I  I  L  I  G  R  *  L  H  A  S  V  Y  V  L  T  S  S  N  L
WS     1981  ATTATACTTATAGGTAGATAACTCCATGCAAGTGTATACGTACTTACAAGTTCAAATTTA  2040
             ||||||||||||||||||||||||||||||||||||||||||||||||||||||||||||
Col-0  1981  attatacttataggtagataactccatgcaagtgtatacgtacttacaagttcaaattta  2040
               I  I  L  I  G  R  *  L  H  A  S  V  Y  V  L  T  S  S  N  L

               *  Q  C  R  F  G  K  Q  I  R  Y  E  S  R  K  S  T  A  D  T
WS     2041  TAACAATGCAGGTTTGGGAAACAAATTAGATATGAATCTCGAAAATCTACAGCAGATACG  2100
             ||||||||||||||||||||||||||||||||||||||||||||||||||||||||||||
Col-0  2041  taacaatgcagGTTTGGGAAACAAATTAGATATGAATCTCGAAAATCTACAGCAGATACG  2100
               *  Q  C  R  F  G  K  Q  I  R  Y  E  S  R  K  S  T  A  D  T

               K  K  R  M  K  G  R  F  T  K  A  G  A  D  Y  D  Y  D  P  R
WS     2101  AAGAAAAGAATGAAAGGAAGATTTACAAAAGCTGGTGCTGATTATGACTATGACCCACGA  2160
             ||||||||||||||||||||||||||||||||||||||||||||||||||||||||||||
Col-0  2101  AAGAAAAGAATGAAAGGAAGATTTACAAAAGCTGGTGCTGATTATGACTATGACCCACGA  2160
               K  K  R  M  K  G  R  F  T  K  A  G  A  D  Y  D  Y  D  P  R
```

```
                  A  N  D  I  N  K  G  K  I  Q  T  *  A  N  D  N  Q  A  Q  L
WS      2161   GCTAATGATATTAACAAAGGGAAAATACAAACATGAGCTAATGATAACCAAGCTCAATTG  2220
               ||||||||||||||||||||||||||||||||||||||||||||||||||||||||||||
Col-0   2161   GCTAATGATATTAACAAAGGGAAAATACAAACATGAgctaatgataaccaagctcaattg  2220
                  A  N  D  I  N  K  G  K  I  Q  T  *  A  N  D  N  Q  A  Q  L

                  *  Y  P  N  K  E  N  K  K  M  V  A  I  I  Y  M  M  I  I  I
WS      2221   TGATATCCAAATAAGGAAAATAAAAAGATGGTTGCAATAATATATATGATGATCATCATC  2280
               ||||||||||||||||||||||||||||||||||||||||||||||||||||||||||||
Col-0   2221   tgatatccaaataaggaaaataaaaagatggttgcaataatatatatgatgatcatcatc  2280
                  *  Y  P  N  K  E  N  K  K  M  V  A  I  I  Y  M  M  I  I  I

                  I  M  V  P  *  F  F  G  L  C  F  A  K  G  V  L  *  F  S  G
WS      2281   ATTATGGTTCCATGATTTTTTGGACTTTGTTTTGCCAAGGGTGTTTTGTAGTTCAGTGGT  2340
               ||||||||||||||||||||||||||||||||||||||||||||||||||||||||||||
Col-0   2281   attatggttccatgattttttggactttgttttgccaagggtgttttgtagttcagtggt  2340
                  I  M  V  P  *  F  F  G  L  C  F  A  K  G  V  L  *  F  S  G

                  F  V  *  L  V  E  I  S  I  S  T  G  C  T  R  L  K  D  C  K
WS      2341   TTTGTCTGATTGGTCGAGATATCGATATCCACTGGATGCACACGCTTAAAGGACTGCAAA  2400
               ||||||||||||||||||||||||||||||||||||||||||||||||||||||||||||
Col-0   2341   tttgtctgattggtcgagatatcgatatccactggatgcacacgcttaaaggactgcaaa  2400
                  F  V  *  L  V  E  I  S  I  S  T  G  C  T  R  L  K  D  C  K

                  G  S  Q  R  G  Q  N  D  L  *  F  S  T  I  V  F  *  F  H  S
WS      2401   GGTTCACAGAGAGGTCAAAACGATCTCTAGTTCTCTACTATAGTGTTTTGATTCCACTCT  2460
               ||||||||||||||||||||||||||||||||||||||||||||||||||||||||||||
Col-0   2401   ggttcacagagaggtcaaaacgatctctagttctctactatagtgttttgattccactct  2460
                  G  S  Q  R  G  Q  N  D  L  *  F  S  T  I  V  F  *  F  H  S

                  I  D  F  F  P  K  L  F  F  S  F  I  I  Y  I  Y  L  F  L  K
WS      2461   ATAGATTTTTTTCCAAAATTATTTTTTTCTTTTATTATTTACATTTATTTATTTCTTAAA  2520
               ||||||||||||||||||||||||||||||||||||||||||||||||||||||||||||
Col-0   2461   atagatttttttccaaaattatttttttcttttattatttacatttatttatttcttaaa  2520
                  I  D  F  F  P  K  L  F  F  S  F  I  I  Y  I  Y  L  F  L  K
```

```
             G  A  L  L  Q  L  L  S  N  Y  F  T  K  N  N  N  *  I  F  R
WS     2521  GGAGCTTTATTGCAGTTACTGTCAAATTATTTTACCAAAAACAATAACTAAATTTTTCGG  2580
             ||||||||||||||||||||||||||||||||||||||||||||||||||||||||||||
Col-0  2521  ggagctttattgcagttactgtcaaattattttaccaaaaacaataactaaatttttcgg  2580
             G  A  L  L  Q  L  L  S  N  Y  F  T  K  N  N  N  *  I  F  R

             *  D  S  N  R  K  L  I  R  F  V  *  F  G  F  C  S  V  K  F
WS     2581  TAAGACTCTAACCGAAAGTTAATTCGGTTCGTTTGATTTGGTTTTTGTTCGGTTAAATTT  2640
             ||||||||||||||||||||||||||||||||||||||||||||||||||||||||||||
Col-0  2581  taagactctaaccgaaagttaattcggttcgtttgatttggtttttgttcggttaaattt  2640
             *  D  S  N  R  K  L  I  R  F  V  *  F  G  F  C  S  V  K  F

             G  L  V  W  F  L  F  D  R  L  S  F  E  *  V  S  V  G  V  A
WS     2641  GGTTTGGTTTGGTTTTTGTTTGATCGATTAAGTTTTGAATGAGTTTCTGTGGGAGTTGCT  2700
             |||||||||||||||||||||||||||||||||||||||||| |||||||||||||||||
Col-0  2641  ggtttggtttggtttttgtttgatcgattaagttttgaatgaatttctgtgggagttgct  2700
             G  L  V  W  F  L  F  D  R  L  S  F  E  *  I  S  V  G  V  A

             S  Q  R  L  K  P  A  T  W  Q  V  F  F  L  L  L  K  P  L  S
WS     2701  TCTCAAAGACTTAAACCTGCAACTTGGCAAGTTTTTTTTCTTCTTCTCAAACCATTGTCT  2760
             ||||||||||||||||||||||||||||||||||||||||||||||||||||||||||||
Col-0  2701  tctcaaagacttaaacctgcaacttggcaagttttttttcttcttctcaaaccattgtct  2760
             S  Q  R  L  K  P  A  T  W  Q  V  F  F  L  L  L  K  P  L  S

             S  S  Y  S  S  F  K  N  K  C  D  N  N  R  L  V  D  I  Q  *
WS     2761  TCATCATATAGCTCTTTTAAAAATAAATGTGACAATAATCGATTGGTGGACATACAGTAG  2820
             ||||||||||||||||||||||||||||||||||||||||||||||||||||||||||||
Col-0  2761  tcatcatatagctcttttaaaaataaatgtgacaataatcgattggtggacatacagtag  2820
             S  S  Y  S  S  F  K  N  K  C  D  N  N  R  L  V  D  I  Q  *

             N  A  K  K  K  I  A  D  W  R  R  S  K  N  I  *  T  T  S  F
WS     2821  AATGCTAAGAAAAAAATAGCCGATTGGAGAAGAAGCAAGAATATATAAACCACCTCTTTT  2880
             ||||||||||||||||||||||||||||||||||||||||||||||||||||||||||||
Col-0  2821  aatgctaagaaaaaaatagccgattggagaagaagcaagaatatataaaccacctctttt  2880
             N  A  K  K  K  I  A  D  W  R  R  S  K  N  I  *  T  T  S  F
```

```
             A  N  *  R  Y  V  F  V  M  A  F  *  F  F  L  I  T  S  S  L
WS     2881  GCAAATTAAAGATACGTGTTTGTCATGGCCTTTTAATTTTTCCTAATAACAAGTTCTTTA  2940
             ||||||||||||||||||||||||||||||||||||||||||||||||||||||||||||
Col-0  2881  gcaaattaaagatacgtgtttgtcatggccttttaatttttcctaataacaagttcttta  2940
             A  N  *  R  Y  V  F  V  M  A  F  *  F  F  L  I  T  S  S  L

             I  I  S  H  E  T  K  L  N  C  L  R  N  I  L  I  F  P  A  A
WS     2941  ATCATTTCTCACGAAACAAAACTCAACTGTCTTAGAAATATATTAATATTCCCTGCGGCT  3000
             ||||||||||||||||||||||||||||||||||||||||||||||||||||||||||||
Col-0  2941  atcatttctcacgaaacaaaactcaactgtcttagaaatatattaatattccctgcggct  3000
             I  I  S  H  E  T  K  L  N  C  L  R  N  I  L  I  F  P  A  A

             R  I  L  *  H  C  T  C  *  G  Q  L  N  L  R  Y  *  T  L  L
WS     3001  CGGATCTTGTGACACTGCACATGTTGAGGGCAGCTAAATTTACGTTACTAAACGTTGCTT  3060
             ||||||||||||||||||||||||||||| ||||||||||||||||||||||||||||||
Col-0  3001  cggatcttgtgacactgcacatgttgaggacagctaaatttacgttactaaacgttgctt  3060
             R  I  L  *  H  C  T  C  *  G  Q  L  N  L  R  Y  *  T  L  L

             V  S  S  R  L  N  I  L  V  C  N  K  *  *  L  Y  M  E  Y  N
WS     3061  GTTTCGTCTCGTCTCAATATTTTAGTGTGTAATAAGTAATAACTATATATGGAATATAAT  3120
             ||||||||||||||||||||||||||||||||||||||||||||||||||||||||||||
Col-0  3061  gtttcgtctcgtctcaatattttagtgtgtaataagtaataactatatatggaatataat  3120
             V  S  S  R  L  N  I  L  V  C  N  K  *  *  L  Y  M  E  Y  N

             I  *  A  F  K  K  P  S  N  N  N  K  *  K  P  S  L  N  W  T
WS     3121  ATATGAGCATTTAAAAAACCATCGAACAATAATAAATAAAAACCAAGCCTTAATTGGACA  3180
             ||||||||||||||||||||||||||||||||||||||||||||||||||||||||||||
Col-0  3121  atatgagcatttaaaaaaccatcgaacaataataaataaaaaccaagccttaattggaca  3180
             I  *  A  F  K  K  P  S  N  N  N  K  *  K  P  S  L  N  W  T

             S  P  I  *  N  L  T  I  D  Q  N  K  V  R  N  E  I  *  P  S
WS     3181  AGTCCGATTTAAAATCTTACAATTGACCAAAATAAAGTTAGGAACGAGATCTAGCCATCA  3240
             ||||||||||||||||||||||||||||||||||||||||||||||||||||||||||||
Col-0  3181  agtccgatttaaaatcttacaattgaccaaaataaagttaggaacgagatctagccatca  3240
             S  P  I  *  N  L  T  I  D  Q  N  K  V  R  N  E  I  *  P  S
```

```
                F  T  K  H  *  I  N  D  L  L  L  S  F  I  I  *  *  L  Y  H
WS      3241  TTTACTAAACATTAAATTAATGATTTACTTTTGTCATTTATTATATAATAATTATATCAT  3300
              ||||||||||||||||||||||||||||||||||||||||||||||||||||||||||||
Col-0   3241  tttactaaacattaaattaatgatttacttttgtcatttattatataataattatatcat  3300
                F  T  K  H  *  I  N  D  L  L  L  S  F  I  I  *  *  L  Y  H

                H  K  S  S  R  W  S  I  *  Y  S  L  Y  K  T  *  H  V  S  C
WS      3301  CATAAATCTAGCAGATGGAGTATTTAATACTCGTTATACAAAACATAACATGTATCTTGC  3360
              |||||||||||||||||||||||| |||||||||||||||||||||||||||||||||||
Col-0   3301  cataaatctagcagatggagtatttgatactcgttatacaaaacataacatgtatcttgc  3360
                H  K  S  S  R  W  S  I  *  Y  S  L  Y  K  T  *  H  V  S  C

                I  L  A  N  *  *  P  K  V  E  K  N  S  I  L  L  W  Q  N  *
WS      3361  ATTCTTGCTAATTAATGACCAAAAGTAGAAAAGAATTCTATATTGTTATGGCAAAATTAA  3420
              ||||||||||||||||||||||||||||||||||||||||||||||||||||||||||||
Col-0   3361  attcttgctaattaatgaccaaaagtagaaaagaattctatattgttatggcaaaattaa  3420
                I  L  A  N  *  *  P  K  V  E  K  N  S  I  L  L  W  Q  N  *

                N  L  K  N  D  L  Y  C  F  F  L  L  S  K  N  V  S  I  V  N
WS      3421  AATTTAAAAAACGATCTCTATTGTTTCTTTTTGTTAAGTAAAAATGTCTCTATTGTTAAT  3480
              ||||||||||||||||||||||||||||||||||||||||||||||||||||||||||||
Col-0   3421  aatttaaaaaacgatctctattgtttctttttgttaagtaaaaatgtctctattgttaat  3480
                N  L  K  N  D  L  Y  C  F  F  L  L  S  K  N  V  S  I  V  N

                K  K  T     R  F  F  V  *  E  F  F  L  L  L  Y  G  T  K  *  I
WS      3481  AAAAAAA--CTCGCTTTTTCGTTTAAGAATTTTTTTTATTGTTGTATGGAACTAAGTGAA  3538
              |||||||  |||||||||||||||||||||||||||||||||||||||||||||||||||
Col-0   3481  aaaaaaaaactcgctttttcgtttaagaattttttttattgttgtatggaactaagtgaa  3540
                K  K  K  L  A  F  S  F  K  N  F  F  Y  C  C  M  E  L  S  E

                 E  N  N  L  T  C  V  E  *  *  I  P  L  P  N  I  *  A  S  P
WS      3539  TTGAGAATAATTTGACATGTGTCGAGTAATAGATTCCTTTACCAAATATATAAGCATCTC  3598
              ||||||||||||||||||||||||||||||||||||||||||||||||||||||||||||
Col-0   3541  ttgagaataatttgacatgtgtcgagtaatagattcctttaccaaatatataagcatctc  3600
                L  R  I  I  *  H  V  S  S  N  R  F  L  Y  Q  I  Y  K  H  L
```

```
                I  N  *  D  V  H  Y  F  N  L  K  K  Y  K  V  Q  E  T  Y  Y
WS     3599  CAATTAATTAAGATGTACATTACTTTAATTTGAAAAAATACAAAGTACAAGAAACGTATT  3658
             ||||||||||||||||||||||||||||||||||||||||||||||||||||||||||||
Col-0  3601  caattaattaagatgtacattactttaatttgaaaaaatacaaagtacaagaaacgtatt  3660
             Q  L  I  K  M  Y  I  T  L  I  *  K  N  T  K  Y  K  K  R  I

                *  Y  N  L  L  D  S  F  F  I  Y  A  L  A  S  V  P  N  H  N
WS     3659  ACTAGTATAATTTATTAGACTCCTTCTTTATTTACGCCTTAGCTTCCGTGCCTAATCACA  3718
             ||||||||||||||||||||||||||||||||||||||||||||||||||||||||||||
Col-0  3661  actagtataatttattagactccttctttatttacgccttagcttccgtgcctaatcaca  3720
             T  S  I  I  Y  *  T  P  S  L  F  T  P  *  L  P  C  L  I  T

                T  S  Y  L  H  I  V  *  S  A  L  I  P  Y  T  K  T  I  F  N
WS     3719  ATACTAGTTATTTACATATAGTTTAGTCAGCATTAATTCCTTACACTAAAACCATTTTTA  3778
             ||||||||||||||||||||||||||||||||||||||||||||||||||||||||||||
Col-0  3721  atactagttatttacatatagtttagtcagcattaattccttacactaaaaccattttta  3780
             I  L  V  I  Y  I  *  F  S  Q  H  *  F  L  T  L  K  P  F  L

                M  T  P  L  L  S  L  *  S  T  L  L  L  X
WS     3779  ACATGACTCCATTGCTCTCCCTCTGATCAACCCTCCTCCTCC  3820
             ||||||||||||||||||||||||||||||||||||||||||
Col-0  3781  acatgactccattgctctccctctgatcaaccctcctcctcc  3822
             T  *  L  H  C  S  P  S  D  Q  P  S  S  S
```

Figure S4: Alignment details of OMG1 in WS vs Col-0 Cultivar. Alignment of OMG1 genomic DNA in WS cultivar and Col-0 cultivar shows that sequence is identical in the upstream ATG region and coding region (highlighted in yellow). All differences between the alignments are highlighted in green. A point mutation is observed at position 2197and 3327 along with a 2 basepair deletion in WS observed at position 3488.

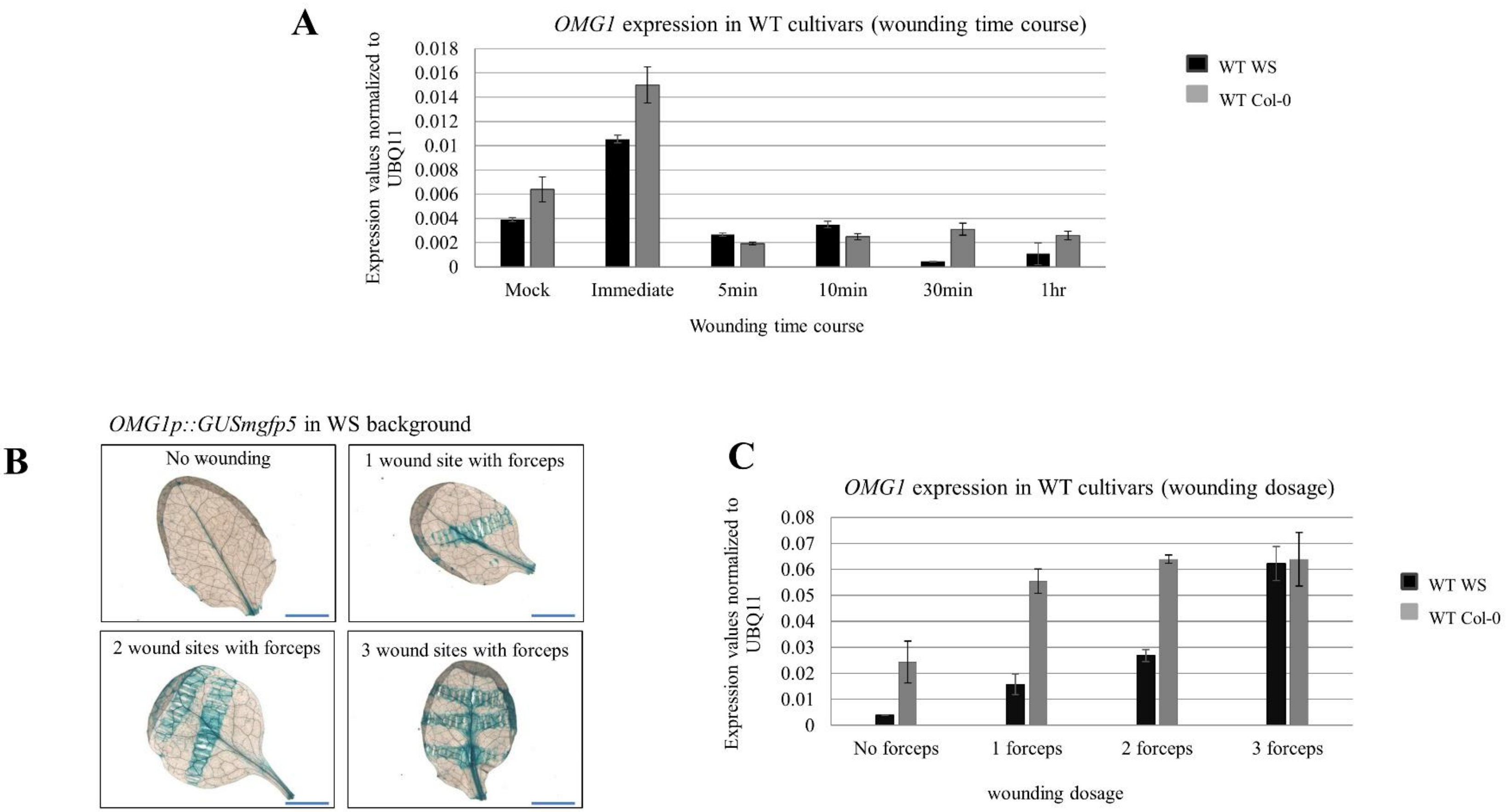

Figure S5. Characterization of *OMG1* expression in WT WS and WT Col-0 with qRT-PCR analysis upon wounding experiments. A) qRT-PCR shows that *OMG1* expression (normalized to UBQ11) is consistently upregulated immediately after wounding in both WS and Col-0 cultivars and diminishes rapidly. Black bars represent the wild type (WT) WS cultivar and the grey bars represent the WT Col-0 cultivars. **B)** Histochemical GUS stained transgenic *OMG1* 5'flanking region::GUS plants illustrates the wounding dosage experiment done in Fig. S2c . Forceps were used to wound the leaves to create a wounding response of increasing degrees. Scale bars: 100 µm. **C)** The WS and Col-0 cultivar respond to the wounding dosage differently. In Col-0, once wounded OMG1 expression researches a threshold and plateaus regardless of the wounding dosage, whereas in WS OMG1 expression increases with the wounding dosage. All error bars represent the standard error of the mean of triplicate representative experiments.

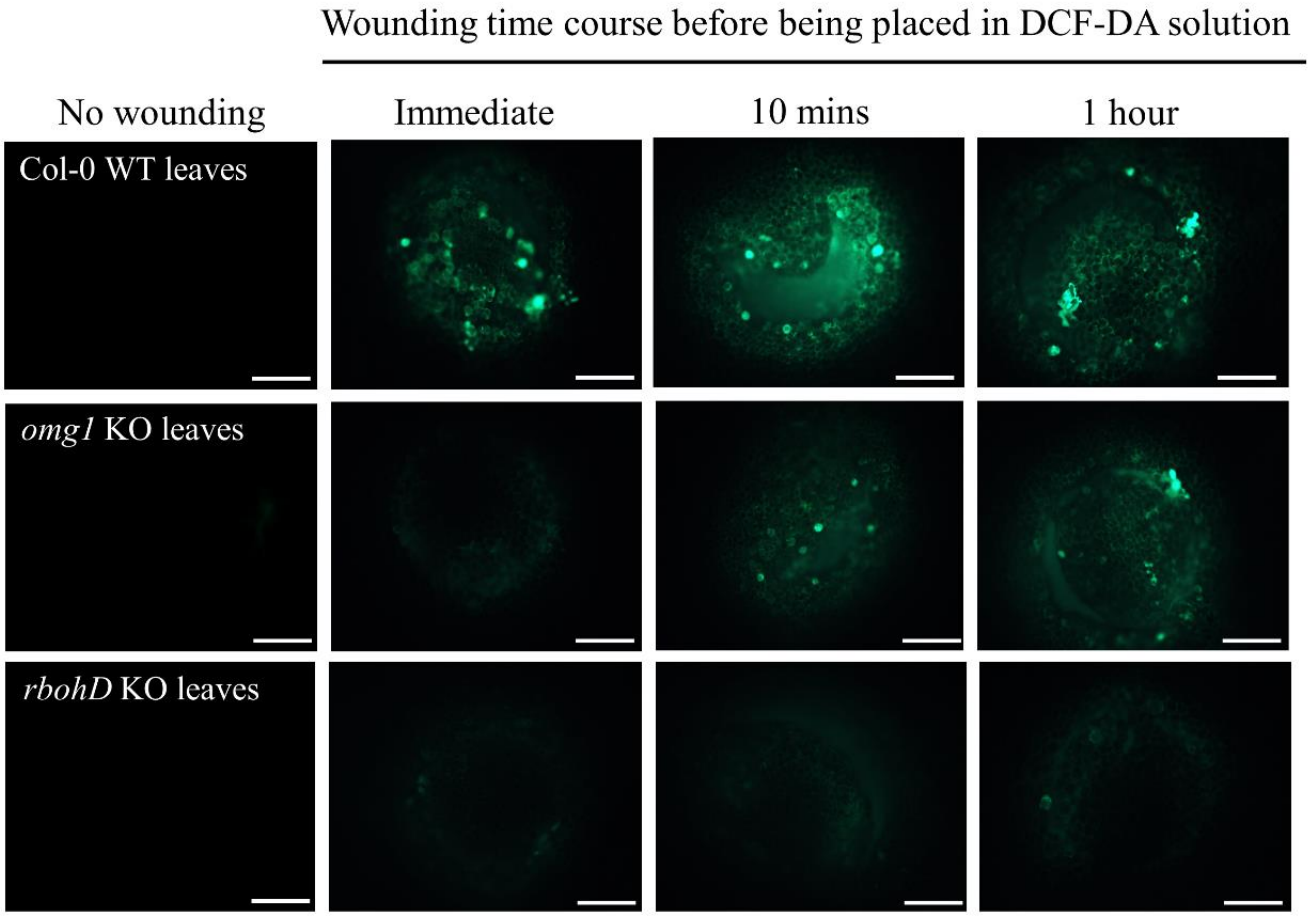

Figure S6: Time course of the DCF-DA fluorescent ROS assay described in Figure 5. A pipette tip was used to wound the surface of the leaves from the wild-type (WT) line, *omg1* KO line and *rboh D* KO line (a known ROS deficient mutant). Upon wounding, ROS is usually produced around the site of injury. The DCF-DA reagent is then oxidized by ROS resulting in appearance of green fluorescence. Plants were wounded and placed immediately, 10 mins post wounding, or 1 hr post wounding into the DCF-DA solution for 20 minutes before visualized on the light microscope. In the WT plants, green fluorescence around the wound site i was observed at all time points post wounding. Whereas, in the *omg1* KO line, the accumulation of ROS was only seen after 10 mins post wounding. However, in the *rboh D* KO line ROS production was not detected. Images were taken on the Olympus BX51 compound scope at 10x magnification, exposure time 55ms. Scale bars: 100 µm.
